# Assessing the Potential of the MTG-FCI Geostationary Mission for the Detection of Methane Plumes

**DOI:** 10.1021/acs.est.5c07974

**Published:** 2026-02-16

**Authors:** Shanyu Zhou, Javier Gorroño, Javier Roger, Itziar Irakulis-Loitxate, Rasmus Lindstrot, Zhipeng Pei, Lulu Si, Luis Guanter

**Affiliations:** † Research Institute of Water and Environmental Engineering (IIAMA), 16774 Universitat Politècnica de València, 46022 València, Spain; ‡ International Methane Emissions Observatory, United Nations Environment Program, 75015 Paris, France; § 84514 EUMETSAT, 64295 Darmstadt, Germany; ∥ State Key Laboratory of Information Engineering in Surveying, Mapping and Remote Sensing, 12390 Wuhan University, Wuhan 430072, China; ⊥ Environmental Defense Fund, Amsterdam 1083 HN, The Netherlands

**Keywords:** MTG-FCI, methane plume detection, earth observation, geostationary remote sensing, shortwave infrared

## Abstract

The Flexible Combined Imager aboard the Meteosat Third Generation satellite (MTG-FCI) provides geostationary observations over Europe and Africa, the Middle East, parts of South America, and the surrounding waters. The FCI samples the visible, near-infrared, and shortwave infrared spectral windows with a spatial resolution at nadir between 500 and 1000 m, and a 10 min temporal sampling interval. This configuration offers a potential for methane retrievals using multiband multipass retrieval (MBMP) methods, as shown with other multispectral missions. The potential of the MTG-FCI system for the detection and monitoring of single methane plumes is evaluated in this article through different approaches. End-to-end simulations using high-resolution WRF-LES methane plumes over Algeria showed that MTG-FCI can detect emissions as low as 30 t/h, where initial plume signals become visible, with a clearer detection above 50 t/h. Additionally, mass-balance modeling estimated a minimum detection limit of 20–30 t/h across the central MTG-FCI disk (GSD ≤ 1 km) under optimal conditions. We illustrate the use of the MTG-FCI for the monitoring and quantification of methane plumes using a real transient emission detection from a compressor station in Algeria (34.676° N, 6.191° E) on September 29, 2023, capturing its full evolution from 10:48 to 15:58 UTC. This event corresponded to an emission rate of 389 ± 81 t/h and a total methane release of ∼320 tons, with results broadly consistent with independent satellite estimates. These findings highlight MTG-FCI’s ability to track large transient methane plumes in near real-time, complementing polar-orbiting sensors.

## Graphical abstract

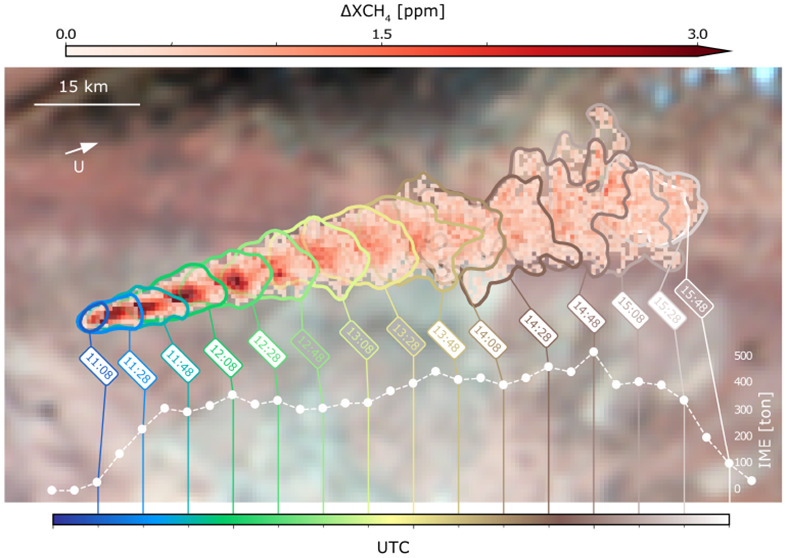

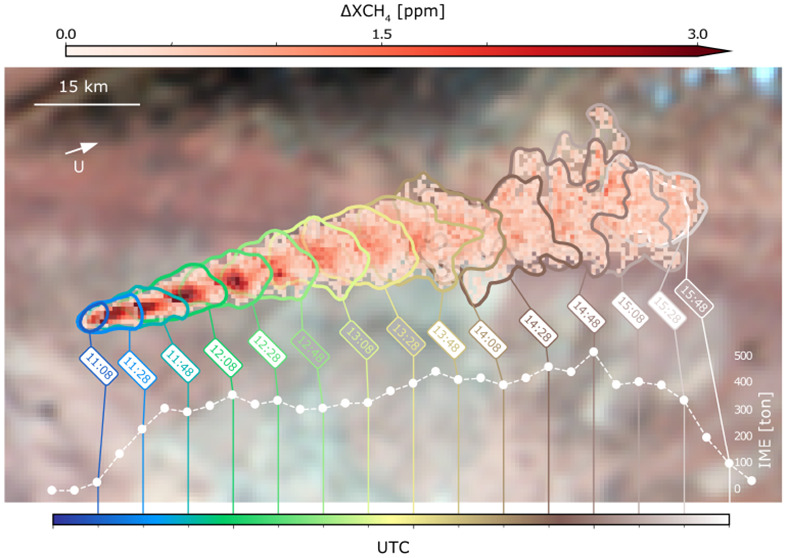

## 1 Introduction

Methane (CH_4_) is a potent greenhouse gas with a global warming potential (GWP) approximately 86 times greater than carbon dioxide (CO_2_) over a 20-year period.^1^ Despite its relatively short atmospheric lifetime of about nine years,^2^ methane substantially contributes to climate change due to its strong radiative forcing effect. Rapid reductions in methane emissions can lead to near-term climate benefits.^3^ Anthropogenic methane emissions primarily originate from the oil and gas (O&G) industry, coal mining, agriculture, and waste management.^4,5^ Among these, O&G emissions, often occurring as super emissions from discrete point sources, present a promising target for mitigation due to the availability of cost-effective reduction strategies.^6^

The detection and quantification of large methane emissions from point sources have been significantly strengthened by satellite-based spectrometers operating in the 1600–2500 nm range (atmospheric window) with contiguous spectral sampling. The TROPOspheric Monitoring Instrument (TROPOMI) aboard the Sentinel-5 Precursor provides daily global coverage with high spectral sensitivity, enabling the detection of large methane plumes around the globe.^7^ Hyperspectral imaging missions such as EMIT, PRISMA, and EnMAP retrieve point-source methane plumes by resolving fine absorption features and applying physically based spectral inversion methods,^8–10^ enabling detailed plume characterization at moderate spatial resolutions. At a finer facility-scale resolution, GHGSat offers targeted monitoring of methane emissions from individual sites.^11^

Multispectral missions offer an alternative approach to methane detection by utilizing a limited number of discrete spectral bands with sensitive to methane absorption. Instruments such as the Sea and Land Surface Temperature Radiometer (SLSTR) aboard Sentinel-3, the Visible Infrared Imaging Radiometer Suite (VIIRS) aboard Suomi-NPP and the Joint Polar Satellite System (JPSS; NOAA-20 and NOAA-21), and the Multispectral Imager (MSI) on Sentinel-2 have already demonstrated potential in detecting methane emissions through the analysis of near-infrared (NIR) and shortwave infrared (SWIR) reflectance variations.^12–16^ These sensors operate in sun-synchronous orbits, providing global coverage with revisit times ranging from several days to subdaily observations. Sentinel-2A, 2B, and the recently launched 2C revisit the same location approximately every 5 days at the equator, offering high spatial resolution (20 m) in the visible-to-SWIR range, with shorter intervals at higher latitudes. Sentinel-3A and 3B provide near-daily global coverage at a moderate spatial resolution (500 m). VIIRS offers subdaily observations at a 750 m spatial resolution, providing an operational, high-temporal-frequency multispectral capability that has recently shown methane plume detection under favorable surface reflectance conditions. However, the detection capabilities of these sensors are constrained by common factors such as cloud cover^14^ and surface reflectance variability.^15^ Moreover, their coarse spectral resolution compared to hyperspectral instruments leads to a lower sensitivity to spectral features of methane.^13^ Despite demonstrated effectiveness, these satellites operate in low Earth orbit, limiting their revisit frequency, thus restricting their ability to fully monitor transient emissions.^17^

Regarding temporal resolutions, the best performance is the one offered by geostationary satellites such as the Advanced Himawari Imager (AHI) aboard Himawari-8/9 and the Advanced Baseline Imager (ABI) aboard the GOES satellite series, which provides continuous monitoring of the Americas and Asia-Pacific regions at 5 to 10 min intervals with a ground sampling distance (GSD) of 1 to 2 km at nadir.^18,19^ The GOES-16 and GOES-17 satellites, primarily designed for weather and environmental monitoring, have demonstrated their capability to detect large methane emission events through reflected solar radiation in the SWIR and NIR channels. Recent studies have leveraged GOES-ABI data to detect methane plumes over North America, showing that geostationary observations can provide valuable insights into emission dynamics with unprecedented temporal coverage,^20^ estimating a detection limit in the range of 10 to 100 tons per hour (t/h). This high-frequency observation capability allows for the identification of short-lived emission events, such as those associated with equipment malfunctions, gas venting, and blowouts in the O&G sector. However, the relatively coarse spatial resolution of GOES-ABI limits its ability to resolve smaller sources.

The Flexible Combined Imager aboard the Meteosat Third Generation satellite (MTG-FCI) represents a significant advancement in methane monitoring, offering high temporal resolution and broad spectral coverage from a geostationary orbit sampling Europe and Africa, the Middle East, parts of South America, and the surrounding waters. The MTG-FCI captures data every 10 min in full disk and 2.5 min in Europe with a GSD of 0.5 to 1 km at nadir. Its spectral configuration includes multiple bands in the NIR and SWIR regions,^21^ encompassing methane-sensitive wavelengths, thereby enabling effective detection of methane enhancements. The MTG-FCI is the first geostationary mission applicable for continuous regional methane monitoring across Europe and Africa, building upon the approach successfully applied to the GOES series over the Americas.

In this study, we investigated the potential of the MTG-FCI for methane detection, focusing on its retrieval performance and detection capability. We performed an end-to-end simulation over one of the most frequently observed methane emission regions in the UNEP’s International Methane Emissions Observatory (IMEO) plume database, located in Algeria, using the multiband multipass (MBMP) method to assess the instrument’s capabilities. Furthermore, we estimated the theoretical minimum detectable methane emission rate for the MBMP retrieval applied to the MTG-FCI, using an empirical formulation^22^ and accounting for key atmospheric and instrumental parameters across the full disk. We also illustrated the potential of the MTG-FCI for the detection and monitoring of transient methane plumes by analyzing a real emission event in Algeria on September 29, 2023.

## 2 Materials and Methods

### 2.1 MTG-FCI System

The FCI is an advanced imaging instrument deployed aboard the Meteosat Third Generation Imager (MTG-I) satellite.^23^ It is designed to provide high-resolution geostationary observations across 16 spectral bands spanning from the visible to the thermal infrared. This enables the monitoring of a wide range of atmospheric and surface phenomena within large parts of Europe and Africa, the Middle East, and parts of South America and the surrounding waters. In particular, its spectral channels at 1.6 μm (NIR1.6) and 2.2 μm (NIR2.2) offer significant potential for methane emission detection. The NIR2.2 spectral channel is available in both the FDHSI (Full Disc High Spectral Resolution Imagery) configuration, with a central GSD of 1 km, and the HRFI (High Spatial Resolution Fast Imagery) configuration, which achieves a nadir GSD of 0.5 km. In contrast, the NIR1.6 spectral channel is recorded exclusively in FDHSI mode.

Figure 1 provides an overview of the spatial and spectral features of MTG-FCI relevant to methane detection. The first panel (Figure 1a) illustrates the approximate effective pixel resolution (Res_eff_) of the HRFI configuration, which varies across the field of view due to the geostationary observation geometry. To account for the varying spatial resolution along and across the track, it is estimated using the geometric mean of the latitudinal and longitudinal ground sampling distances: ${\mathrm{Res}}_{\mathrm{eff}}\approx \sqrt{\mathrm{dLat}\times \mathrm{dLon}}$ , which provides a more representative measure of the pixel footprint at different viewing geometries. As shown in Figure 1b, the MTG-FCI effectively captures wavelengths that are highly sensitive to methane’s spectral absorption features, with a finer spectral resolution than Sentinel-2’s MSI. Notably, methane exhibits strong absorption around the 2.2 μm wavelength, whereas the adjacent 1.6 μm wavelength range experiences minimal absorption. This contrast of absorption properties between two wavelength regions thusallows for the retrieval of methane concentrations.

**Figure 1:**
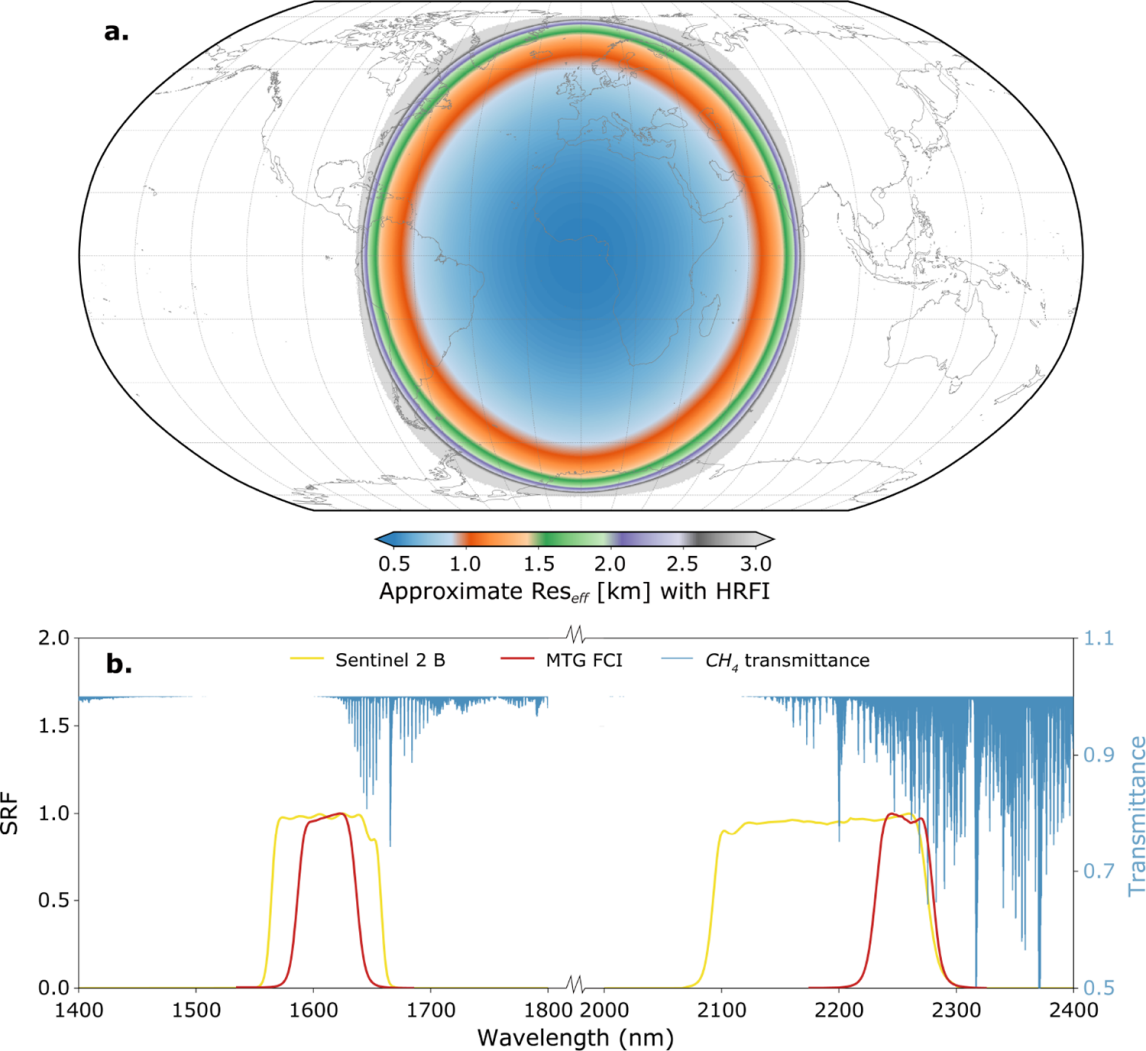
Overview of MTG-FCI spatial and spectral properties relevant for methane detection. (a) Approximate Res_eff_ in kilometers for the HRFI configuration, illustrating spatial resolution variations across the field of view. (b) Averaged FCI Spectral Response Functions (SRF) of methane-related wavelength, Yellow curve: Sentinel-2B; Red curve: MTG-FCI; Blue lines: high-resolution calculated CH_4_ transmittance spectrum.

### 2.2 Methane Retrieval

The retrieval framework developed for the GOES-ABI instrument^20^ and Sentinel-2/MSI^13^ served as a reference in designing the MTG-FCI retrieval scheme. In particular, the MBMP retrieval algorithm from Gorroño et al.^13^ has been adapted for MTG-FCI data, leveraging band ratios to isolate the methane absorption signal from surface reflectance variations. Specifically, the algorithm utilizes a methane-sensitive spectral channel centered at 2250 nm (NIR2.2 in MTG-FCI) alongside a reference channel at NIR1.6, which exhibits minimal methane absorption. To further mitigate surface-related artifacts, the band ratio from a given observation (containing a methane plume) is normalized against a corresponding ratio from a designated reference day (plume-free). Mathematically, this is expressed as

$$T_{\mathrm{plume}}∼\frac{\rho }{\rho_{\mathrm{ref}}}=\frac{{\left(\frac{\rho_{{\mathrm{NIR}}_{22}}}{\rho_{{\mathrm{NIR}}_{16}}}\right)}_{\mathrm{plume}}}{{\left(\frac{\rho_{{\mathrm{NIR}}_{22}}}{\rho_{{\mathrm{NIR}}_{16}}}\right)}_{\mathrm{plume‐free}}}\approx e^{-\mathrm{AMF}\cdot \sigma_{{\mathrm{CH}}_{4}}\cdot \Delta X_{{\mathrm{CH}}_{4}}}$$ 1

where plume and plume-free refer to the presence and absence of methane plumes, respectively. ρ represents the top-of-the-atmosphere (TOA) radiance. The retrieved transmittance *T*_plume_ is related to the methane concentration enhancement (Δ*X*_CH_4__) through the air mass factor (AMF) and the effective methane absorption cross-section (σ_CH_4__), which represents the absorption strength of methane over the MTG-FCI NIR2.2 channel after convolution with the spectral response functions (SRFs) provided by EUMETSAT (https://nwp-saf.eumetsat.int/downloads/rtcoef_info/visir_srf/rtcoef_mtg_1_fci_srf.html). To model methane’s spectral transmittance and establish the relationship between atmospheric methane enhancement and absorption characteristics, a look-up table (LUT) was employed. The LUT was constructed using high-resolution radiative transfer simulations and subsequently convolved with the same MTG-FCI SRFs to ensure consistency with the instrument’s spectral sampling. This approach effectively accounts for atmospheric interactions and radiative transfer effects, ensuring the accurate retrieval of methane enhancements. Although some common principles were adopted from Sentinel-2 MBMP retrievals to enhance methane detection, such as the incorporation of multispectral and multitemporal information, adaptation to the MTG-FCI introduces two specific challenges.

The first challenge arises from MTG-FCI’s high temporal resolution. For a given target scene (ρ), multiple candidate background observations (ρ_ref_) are available, and an effective selection strategy is required to avoid redundancy while ensuring retrieval stability. Meanwhile, surface-related artifacts (e.g., illumination differences and reflectance variability) need to be minimized. We address both aspects through a background selection strategy that selects the closest available clear-sky day within 2 days of the target date, strictly at the same UTC time, while excluding cloudy or rapidly changing scenes. For instance, if the methane-containing target data correspond to 11:08 UTC on September 29, 2023, the background is selected from the clearest available day at 11:08 UTC (e.g., September 28 or 30). It is also important to ensure that the selected days are free from rapid scene changes such as precipitation or intense human activity. This approach simultaneously minimizes illumination and atmospheric variability and avoids redundant (ρ_ref_) selection, thereby improving retrieval stability and reducing surface-related artifacts.

A second challenge stems from spatial resolution differences between the NIR2.2 (0.5 km/1 km) and NIR1.6 (1 km) channels. To ensure spatial consistency, the NIR1.6 band was upsampled to the finer 0.5 km NIR2.2 grid and coregistered using GeFolki optical flow algorithm.^24–26^ Bilinear interpolation was used to preserve gradients and radiometric consistency, avoiding blocky artifacts from nearest-neighbor resampling. This strategy maintains the native resolution of NIR2.2, which coincides with the strongest methane absorption band in SWIR range, and prevents the unnecessary loss of plume detail that would result from downsampling. Interband displacements were found to be typically below 1 pixel (0.5 km at nadir), indicating that the applied upsampling and coregistration procedure provides a practical improvement in band-to-band alignment. However, these small offsets do not rule out residual retrieval errors, and the coregistration is treated as an operational alignment step that may still subtly influence background variability and methane retrieval uncertainty. Detailed descriptions of the coregistration workflow, displacement estimation (interband, interdate, and intertime), and their quantitative evaluation are provided in the Supporting Information (Section S1).

In practice, cloud masking is essential to ensuring retrieval robustness. Cloud-contaminated pixels were removed using a ratio-based thresholding between IR10.5 and NIR0.4, further validated with MSG cloud masks to exclude residual contamination. Detailed diagnostics of the cloud screening procedure are provided in the Supporting Information (Section S2).

The MTG-FCI methane retrieval described above is implemented in Python and is publicly available at https://github.com/LARS-UPV/CH4FCI.

### 2.3 Detection and Quantification of Methane Enhancements

Methane plume detection in this study is based on a thresholding technique, which employs a dual-threshold approach optimized for MTG-FCI observations. Specifically, pixels exceeding a predefined low threshold (e.g., >2σ, where σ refers to the retrieval standard deviation) are included in the plume mask only if they are spatially connected to pixels surpassing a higher threshold (e.g., the 95th percentile). To further refine the detection, we apply a 3 × 3 median filter to suppress high-frequency noise and visually adjust the mask in cases in which atmospheric or sensor-induced artifacts are present.

To quantify the methane plume, we compute the integrated mass enhancement (IME),^27,28^ which represents the excess mass of methane within the detected plume area. The IME is derived using the following equation, adapted for MTG-FCI’s spatial and spectral characteristics:

$$\mathrm{IME}=M_{{\mathrm{CH}}_{4}}\overset{N}{\underset{j=1}{\sum }}\Delta \Omega_{j}A_{j}$$ 2

$$\Delta \Omega_{j}=\frac{H_{\mathrm{col}}\cdot \Delta X_{CH_{4_{j}}}}{10^{6}\times V_{m}}$$ 3

where *M*_CH_4__ = 0.01604 kg mol^–1^ is the molar mass of methane, *A*_*j*_ = *x*_res_ × *y*_res_ (m^2^) denotes the corresponding pixel area, and *N* is the total number of pixels included in the mask. ΔΩ_*j*_ (mol m^–2^) represents the methane column enhancement in the *j*th pixel. *H*_col_ = 8000 m is the assumed atmospheric column height,^27,29^ representing the vertical extent of methane enhancement considered in the calculation. This is consistent with the scale height of dry air (∼8.4 km^30^) and typical tropospheric depths over northern Algeria (∼7–9 km^31^). *V*_m_ = 0.0224 m^3^ mol^–1^ is the molar volume of an ideal gas under standard temperature and pressure. $\Delta X_{CH_{4_{j}}}$ (ppm) is the calculated methane concentration enhancement in the *j*th pixel under the assumption of uniform dispersion within the *H*_col_.

Uncertainties on IME were estimated by propagating three independent contributions: (i) random noise, quantified from the background standard deviation of Δ*X*_CH_4__ and scaled by the plume pixel count; (ii) plume mask sensitivity, assessed from one-pixel dilation and erosion of the detected plume area; and (iii) column height variability, represented by terrain elevation differences within the plume footprint (approximated by σ_*z*_ ∼ 350 m). These terms were combined in quadrature to obtain the total IME uncertainty. Detailed derivations and diagnostic examples are provided in the Supporting Information (Section S3).

### 2.4 Sensitivity Analysis

#### 2.4.1 End-to-End Simulation

To evaluate the capability of the MTG-FCI in methane detection, an end-to-end simulation was conducted using realistic methane plume data derived from the Weather Research and Forecasting Large Eddy Simulation (WRF-LES model). Following the methodology previously applied to Sentinel-2,^13^ the simulated methane plume was incorporated into the background image to assess retrieval performance and detection limits. The base imagery used to generate FCI-like simulated data sets was obtained from preoperational MTG-FCI Level 1 data provided by the European Organisation for the Exploitation of Meteorological Satellites (EUMETSAT).

The simulation process is composed of several key stages. First, high-resolution methane mass fields from the three-dimensional WRF-LES model were vertically integrated into a 2D map and then spatially binned to the MTG-FCI pixel grid, approximating the plume signal at the instrument’s native resolution. At the Algeria location, the viewing zenith angle (∼43°) and solar zenith angle (∼38°) correspond to an effective air mass factor of ∼2.6, meaning that the line-of-sight path is about 2.6 times the vertical column. These angular effects are inherently represented in the AMF parametrization used in the retrieval. The resulting Δ*X*_CH_4__ fields were converted to plume transmittance on a per-pixel basis using a precomputed LUT (parametrized by AMF and wavelength). Band-effective plume transmittances at 2.2 and 1.6 μm were subsequently obtained by convolution with the MTG-FCI SRFs and applied to the background radiances, yielding simulated radiance images with methane absorption.

Figure 2 depicts the transition from a high-resolution WRF-LES-generated methane plume (a) to the spatial configuration of the MTG-FCI (b), highlighting the impact of spatial resampling on methane enhancement levels. In panel (a), the original plume, simulated with a GSD of 25 m, exhibits fine-scale structures with localized enhancement levels reaching up to 10 ppm. However, after resampling to the 500 m resolution of the MTG-FCI (b), the methane enhancement levels are significantly reduced by approximately an order of magnitude across most of the area, making plume detection more challenging. Panel (c) provides a contextual view, in which the synthetic methane plume is superimposed onto the real scene. The zoomed-in region demonstrates how the plume structure is reshaped at coarser resolution, with a coverage area of approximately 18 × 18 pixels, emphasizing the trade-off between spatial coverage and detection sensitivity in MTG-FCI observations.

**Figure 2:**
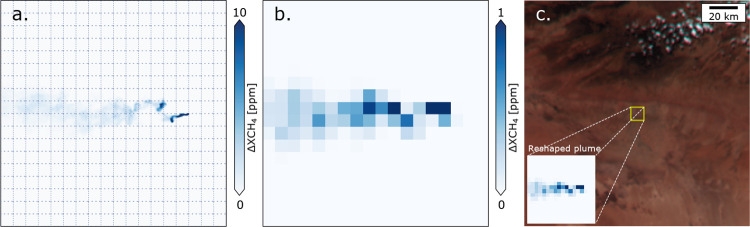
Methane enhancement map (Δ*X*_CH_4__) of the plumes used in the simulations, scaled to a flux rate of *Q* = 50 t/h after resampling to a 500 m resolution. (a) Original Δ*X*_CH_4__ map at 25 m spatial sampling in the range of 0–10 ppm, (b) convolved Δ*X*_CH_4__ map in the range of 0–1 ppm, and (c) schematic diagram of the simulation area overlaying on the MTG-FCI’s RGB image.

#### 2.4.2 Relative Detection Limit Estimation for Comparative Assessment

While end-to-end simulations can yield precise detection limits for specific times and locations, they are computationally intensive and impractical to apply across the entire MTG-FCI disk. Instead, we leverage established empirical formulas to efficiently estimate spatial variations in the minimum detectable methane emission rate (*Q*_min_) across the full MTG-FCI coverage area. Following the formulation proposed by Jacob et al.,^22^ the detection limit for a single pixel can be expressed as

$$Q_{\min}=M_{{\mathrm{CH}}_{4}}\cdot U\cdot \mathrm{GSD}\cdot q\cdot \sigma_{\Delta X_{{\mathrm{CH}}_{4}}}$$ 4

where *M*_CH_4__ is the molar mass of methane. *U* represents the wind speed, derived from the 10 m wind field (*U*_10_) provided by the Global Wind Atlas,^32^ available at a 250 m resolution and resampled to match the FCI pixel grid. GSD is approximately 0.5 km at the nadir and increases with the viewing zenith angle across the disk. $\sigma_{\Delta X_{{\mathrm{CH}}_{4}}}$ corresponds to the uncertainty, defined as the standard deviation of the Δ*X*_CH_4__ estimation. *q* is the number of standard deviations above the noise floor required for confident detection. In this study, we adopt *q* = 2, a commonly used threshold in satellite-based studies of methane detection limits.^22,33^

In practice, directly estimating $\sigma_{\Delta X_{{\mathrm{CH}}_{4}}}$ at the pixel level based on sufficient statistical sampling across multiple observations would be computationally infeasible over the full MTG-FCI disk due to the massive data volume involved. To address this, we approximate the pixel-level uncertainty by computing the standard deviation of Δ*X*_CH_4__ within 300 × 300 pixel blocks (∼150 × 150 km at nadir). This block size provides a balance between computational feasibility and spatial representativeness, and the resulting statistic serves as a representative proxy for all pixels in the block. This provides a conservative approximation of pixel-level uncertainty, consistent with the fact that plume detection typically relies on coherent enhancements across multiple connected pixels, while the native GSD is retained from the instrument geometry. Importantly, this block-based statistic also captures background variability from surface reflectance and atmospheric conditions, meaning that regions with heterogeneous or darker surfaces (e.g., croplands, forests, and mountains) yield elevated values of $\sigma_{\Delta X_{{\mathrm{CH}}_{4}}}$ and thus higher *Q*_min_. In selected regions (e.g., Hassi Messaoud), we additionally conducted finer-scale analyses applying a 10 × 10 pixel block window, which balances statistical significance with improved resolution of plume-scale variations in detection capability.

The estimation of *Q*_min_ was computed based on observations overpassed at 13:38 UTC on three cloud-free days per month during the period from September 2024 to February 2025 (see Section 2.5), resulting in a total of 18 sampled days. Although *Q*_min_ could, in principle, be derived for every clear-sky day, this subsampling strategy was adopted to balance computational feasibility with temporal representativeness, which limits the data volume while still capturing a range of observing conditions. The lowest value among all sampled days was retained to represent the best detection sensitivity achievable under typical conditions.

It is important to note that *Q*_min_ is a relative sensitivity indicator rather than an absolute detection threshold. Actual detectability depends on the spatial coherence and plume morphology, which are not explicitly captured in eq 4.

### 2.5 Satellite and Auxiliary Data

This study integrates multiple satellite and auxiliary data sets to ensure robust methane detection, retrieval, and validation. The primary data set is obtained from the MTG-FCI instrument. To complement these observations, additional data sets from VIIRS, WRF simulations, and reference databases were incorporated for validation, source attribution, and performance benchmarking.

#### 2.5.1 VIIRS Observations

The Visible Infrared Imaging Radiometer Suite (VIIRS), onboard Suomi-NPP, JPSS-1, and JPSS-2 satellites (the latter two are also known as NOAA-20 and NOAA-21) , provides radiance measurements at a resolution of 750 m at nadir. VIIRS SWIR bands M10 (1.6 μm) and M11 (2.2 μm), which are sensitive to methane detection,^16^ were used to trace methane plumes using the same MBMP retrieval framework as for the MTG-FCI. Multiple VIIRS overpasses on September 29, 2023, were used for plume detection, with plume-free scenes from September 28 and 30 as reference. The preprocessing steps included level 1B radiance extraction, spatial subsetting over the ROI, and reprojection for scene alignment. To enable a direct comparison with the MTG-FCI, we additionally derived IME values from the VIIRS retrievals. Since Suomi-NPP and NOAA-20/21 differ in calibration and spectral response,^34,16^ a background bias correction was applied in nonplume areas to harmonize the NOAA-20/21 retrievals with the Suomi-NPP baseline, ensuring consistency across VIIRS onboard platforms.

#### 2.5.2 Additional Data during Processing

The WRF-LES-simulated methane plumes were injected into real FCI backgrounds to conduct end-to-end sensitivity analysis.^28^ For both retrieval and simulation, cloud-affected pixels were excluded using a combined method: a reflectance-ratio-based cloud mask (IR10.5/NIR0.4) and independent cloud flags from MSG. This reduced false positives due to cloud contamination and improved retrieval reliability. The injected plumes correspond to an emission rate (*Q*) of 10 to 90 t/h in steps of 20 t/h, representative of large-scale emission events. The initial wind speed prescribed in the simulation was 5 m/s; however, during the selected snapshot used for retrieval evaluation, the near-surface wind speed across the simulation grid was approximately 3–4 m/s. These conditions reflect a favorable, yet realistic, scenario for geostationary methane plume detection.

#### 2.5.3 Reference Data Sets for Source Attribution

To support the spatial attribution of detected methane plumes, the Oil and Gas Infrastructure Mapping (OGIM) data set^35^ was used to identify known anthropogenic sources such as compressor stations and flaring sites near the observed plumes. The IMEO Plume List (available at Eye on Methane data platform, https://methanedata.unep.org/), curated by the UNEP’s International Methane Emissions Observatory, provided a benchmark reference for known high-emission locations and typical flux magnitudes observed globally. These data sets helped corroborate retrieved results and assess source plausibility.

The estimated overpass times of all data sets used for both retrieval and background reference are summarized in Table 1.

**Table 1:** Satellite Overpass Times for Methane Plume and Reference Imagery from the FCI and VIIRS Sensors Used in This Study

Platform	Plume date/UTC	Reference date/UTC
MTG-FCI (sim.)	2023–09–29/13:18	2023–09–30/13:18
MTG-FCI (real)	2023–09–29/10:48–15:58	2023–09–30/10:48–15:58
VIIRS JPSS-1	2023–09–29/12:24	2023–09–28/12:43
VIIRS JPSS-2	2023–09–29/12:47	2023–09–30/12:28
VIIRS Suomi-NPP	2023–09–29/11:34, 13:14	2023–09–30/12:56

## 3 Results and Discussion

### 3.1 General Retrieval Performance

#### 3.1.1 End-to-End simulation Assessment

Figure 3 presents a set of methane plume simulations on FCI data and their corresponding radiative impact using WRF-LES modeling and MBMP retrieval. The RGB composite in panel (a) delineates the study region in Algeria on September 29, 2023, where high-resolution modeling of methane dispersion was conducted within the marked simulation area. The statistical distribution shown in panel (b) represents enhancement levels under plume-free conditions, indicating the background variability in the absence of simulated methane. The standard deviation (σ = 0.15 ppm) of this distribution defines a detection threshold, as enhancements exceeding 2σ are generally considered distinguishable from background noise.^22,33^

**Figure 3:**
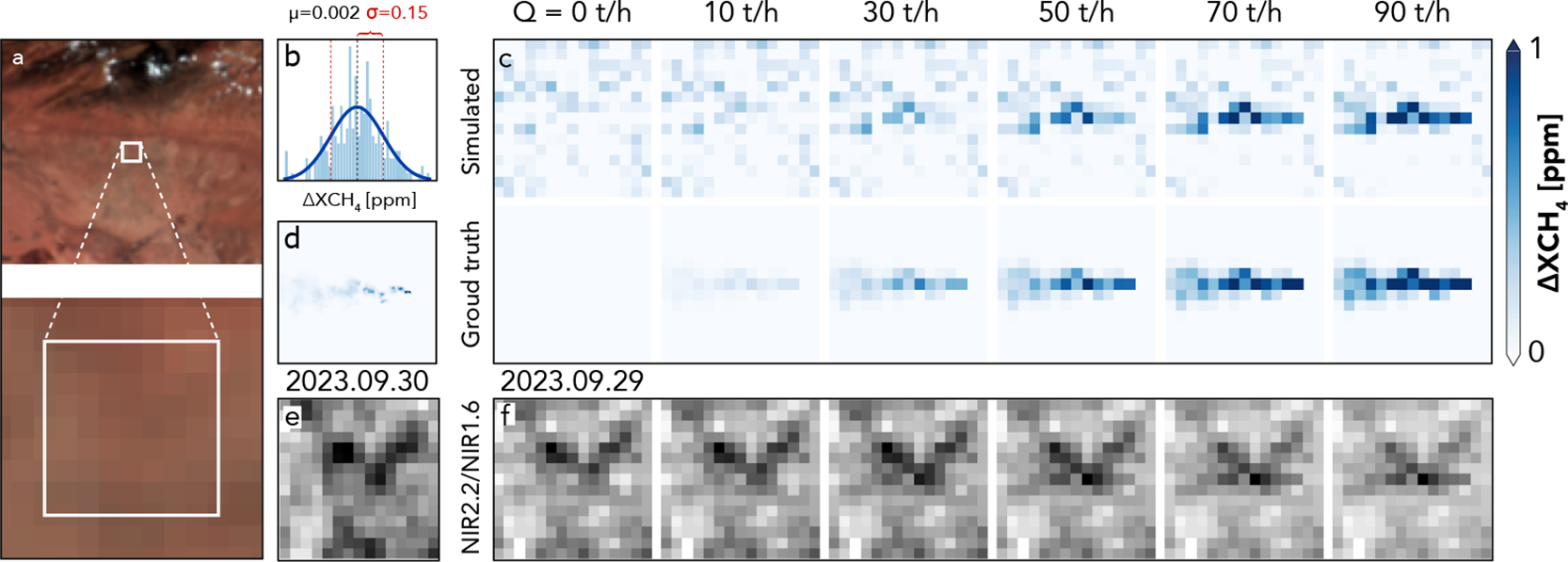
Overview of simulation inputs and outputs for assessing detection sensitivity: (a) RGB composite of the study region, with the white box indicating the simulation area. (b) Statistical distribution of the enhancement map under plume-free conditions (no simulated methane). (c) Simulated Δ*X*_CH_4__ (ppm) at different emission rates, where the top row represents simulated observations, and the bottom row shows the WRF-LES ground truth after convolution to ∼500 m resolution. (d) High-resolution WRF-LES methane distribution (Δ*X*_CH_4__, ppm) at 25 m resolution. (e) Background radiance ratio (NIR2.2/NIR1.6) used in the retrieval. (f) Simulated variations in the radiance ratio for different methane emission rates.

Panel (c) illustrates the simulated Δ*X*_CH_4__ in ppm across different emission rates, from 10 to 90 t/h in steps of 20 t/h. The top row corresponds to real FCI data, including simulated plumes, whereas the bottom row presents the ground truth from the WRF-LES simulation after convolution to ∼599 m resolution (the typical GSD in the Algeria area, obtained from Figure 1a). The results indicate that for emission rates below 30 t/h, enhancement signals remain weak and are difficult to distinguish from background variability. However, starting from 30 to 50 t/h, MTG-FCI retrievals show noticeably enhanced plumes with greater spatial coherence, with enhancement signals exceeding approximately 2σ above background variability, making detection increasingly reliable. This suggests that MTG-FCI’s methane detection capability becomes effective within this emission range, where enhancement signals rise above background noise levels and allow for clear plume identification. The original high-resolution methane distribution from WRF-LES at a 25 m resolution (panel d) provides insight into the fine-scale turbulent structures of the plume, which are partially lost in the lower-resolution retrievals due to spatial averaging effects. Panels (e) and (f) further illustrate the spectral sensitivity to methane enhancement using the ratio of NIR2.2 to NIR1.6 radiances. Panel (e) shows the background radiance ratio under plume-free conditions, while panel (f) highlights how this radiance ratio changes in response to different methane emission rates. These ratios are presented solely to illustrate the spectral sensitivity of the two bands, while the retrieval itself relies on reference-normalized ratios. The increasing contrast with the emission strength supports the use of the NIR2.2/NIR1.6 ratio for plume detection.

These results indicate that the MBMP methods effectively identify methane plume signals, with current detection limits in Algeria estimated at 30–50 t/h under certain illumination and wind conditions (∼3 and 4 m/s on the simulation date). These results reflect best-case real-world scenarios, as the Algerian desert offers a homogeneous, dry, and highly reflective surface and the relatively low wind speeds favored plume accumulation and visibility, which enhance detectability by reducing plume dispersion.

#### 3.1.2 Regional Detection Capability of MTG-FCI

To evaluate the spatial variation in detection sensitivity across the full MTG-FCI disk, we also assessed the minimum detectable methane emission rate (*Q*_min_) under typical observational conditions. To ensure statistical significance, we selected data from 3 days per month between the release of the MTG-FCI data in September 2024 and February 2025 (see details in Supporting Information Table S4), using observations at 13:38 UTC for each selected day. The minimum *Q*_min_ value was determined across all sampled data points from September 2024 to February 2025, representing the lowest detection threshold achievable under these conditions.

The results presented in Figure 4 highlight the capabilities of the MTG-FCI for detecting and monitoring methane emissions at high spatial and temporal resolution. For visualization, Figure 4a provides an overview of regional detection capabilities at a 150 km grid resolution with statistics over a 300 × 300 pixel grid, while Figure 4b utilizes a finer 10 × 10 pixel grid (and 5 km grid size) to illustrate more detailed spatial variations in detection sensitivity. In ideal scenarios, such as over the Sahara, *Q*_min_ can reach 20 t/h, whereas the average value across the domain is considerably higher due to less favorable observational conditions. Notably, the resolution is highest near the satellite’s nadir (Figure 1a), reaching subkilometer scales, while it degrades toward the periphery, where the GSD exceeds 3 km. This variation has significant implications for methane detection, as higher resolution in central regions allows for a finer-scale plume identification, reducing the risk of spatial averaging that can obscure localized emissions. At higher viewing angles, methane enhancement signals weaken due to geometric distortion and the longer atmospheric path, making isolated plumes harder to detect. The coarser effective resolution at oblique views further introduces uncertainties from pixel mixing and parallax, which can reduce retrieval accuracy and complicate emission quantification.^36,37^

**Figure 4:**
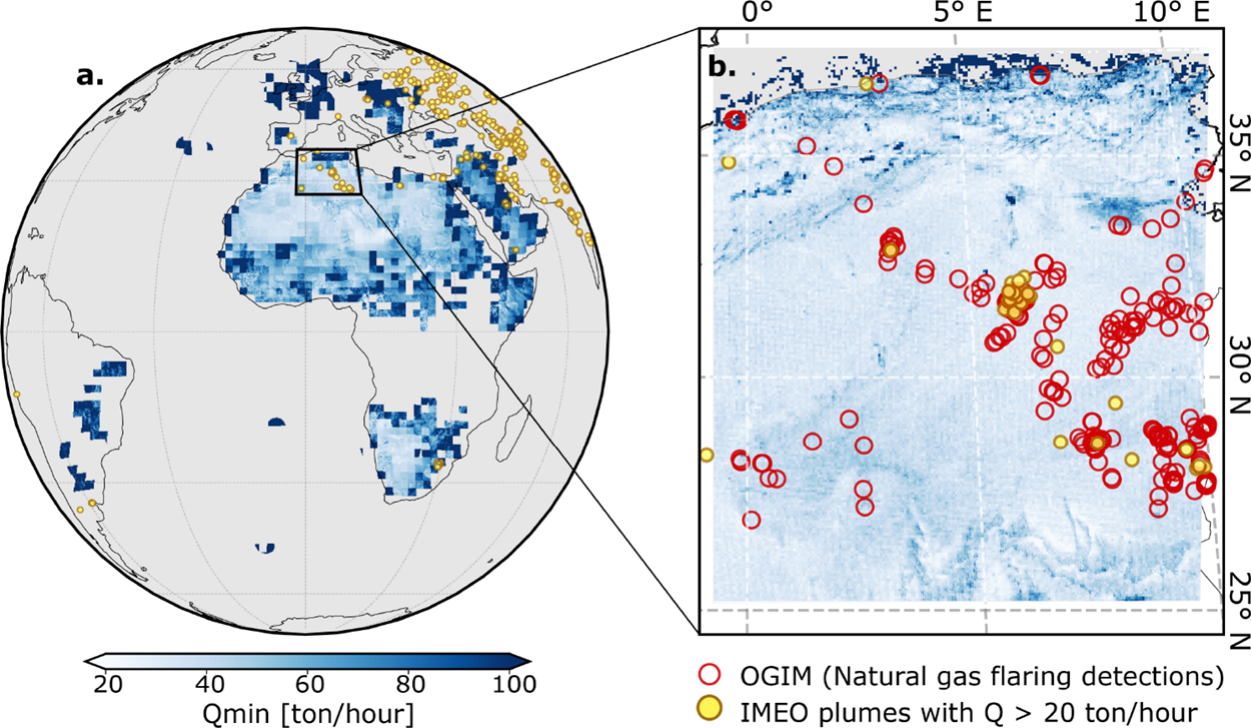
Overview of MTG-FCI methane detection capabilities and regional emission characterization. (a) Spatial distribution of the minimum detectable methane emission rate (*Q*_min_, t/h) across the MTG-FCI coverage area from September 2024 to February 2025. Gray regions indicate cloud-masked areas derived from MSG observations. (b) Methane plume detections over the Hassi Messaoud region overlaid with detected large-scale plumes (*Q* > 20 t/h, yellow) from the IMEO database and natural gas flaring locations from OGIM (red circles). This region is highlighted due to the substantial methane emissions observed during the analysis period.

Beyond viewing geometry, surface properties further modulate the detection capability. The spatial distribution of *Q*_min_ (t/h) across the full MTG-FCI disk, together with detected large-scale plumes (*Q* > 20 t/h) from the IMEO database (Figure 4a, yellow-filled points), reveals clear regional differences: the highest sensitivity is concentrated in North Africa, where strong emission sources such as O&G infrastructure, industrial sites, and landfills are prominent. The apparent gaps shown as gray areas primarily result from persistent cloudiness during the study period (September 2024 to February 2025), as well as from the limited temporal scope of the detection limit estimations. The statistical variability in *Q*_min_ between neighboring grids may also arise from cloud contamination, which inflates detection thresholds and introduces spatial discontinuities. In addition, low-albedo and heterogeneous surfaces such as croplands, forests, and mountainous terrain (appearing as elevated *Q*_min_ values) exhibit more substantial background variability and lower signal-to-noise ratios, further limiting detectability relative to bright homogeneous surfaces, e.g., deserts.

Figure 4b zooms into the Hassi Messaoud region, a major O&G extraction hub in North Africa, recognized for its significant methane releases.^38^ Plume footprints from the IMEO database are overlaid with flaring locations from OGIM, providing context for the potential emission sources. Despite MTG-FCI’s ability to achieve a minimum *Q*_min_ of approximately 20–30 t/h in its central coverage area, the observed methane plume activity over North Africa appears relatively sparse. While significant plumes are identified in regions like Hassi Messaoud, most of the continent shows relatively few strong emissions (>10 t/h), indicating that MTG-FCI’s detection threshold may be insufficient for smaller or more diffuse sources, reinforcing its role as an effective tool for capturing major transient emission events rather than continuous low-intensity leaks.

### 3.2 Case Study

To demonstrate the capabilities of the MTG-FCI instrument for monitoring transient methane emissions, we analyzed a prominent case of methane release from a compressor station in Algeria previously shown by de Jong et al.,^16^ located at 34.676°N, 6.191°E. The high temporal resolution of the MTG-FCI data enabled continuous monitoring of the plume’s onset, growth, and dissipation, as illustrated in the time series spanning from 10:48 to 15:58 UTC. To complement the MTG-FCI retrievals, we incorporated VIIRS observations from the Suomi-NPP, JPSS-1, and JPSS-2 satellites into the analysis. These data were processed using the same MBMP retrieval framework (Section 2.2). The VIIRS detections serve as supplementary snapshots during the key period of plume evolution, allowing cross-platform comparison of spatial patterns and retrieval magnitudes. In addition, external plume mass estimates reported by de Jong et al.^16^ were referenced to benchmark our emission quantification results.

A comprehensive visualization of the methane plume development is provided in Figure 5. Panel (a) illustrates the spatiotemporal evolution of the plume using RGB composites and methane enhancement overlays from MTG-FCI and VIIRS, with VIIRS detections highlighted in red-outlined frames. Panel (b) identifies the emission source overlaid on Sentinel-2 imagery acquired on September 30, 2023. Panel (c) presents the temporal trends of IME (black dots, line) and plume size (blue line). On top of it, red crosses indicate our VIIRS-based IME estimates, whereas cyan crosses show the independent estimates from de Jong et al.^16^

**Figure 5:**
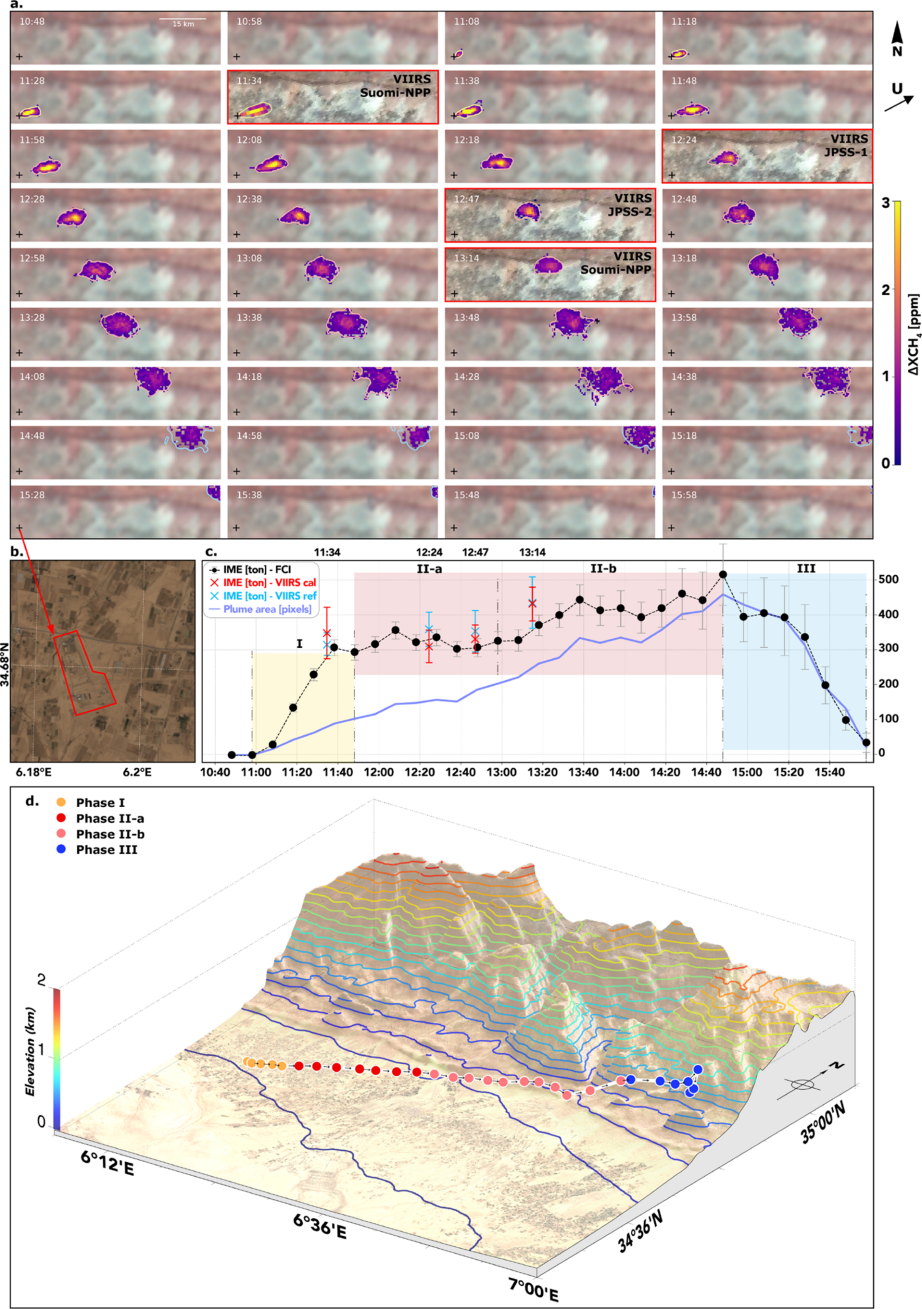
Case study in Algeria. (a) Time series of RGB composites over the emission site between 10:48 and 15:58 UTC on September 29, 2023, showing methane plume retrievals from MTG-FCI and VIIRS (panels with red outlines). For the overpasses after 14:58, the IME mask extends beyond the map displayed. (b) Sentinel-2 image of the site on September 30, 2023, with the emission facility outlined in red. (c) Temporal evolution of IME (black dotted line with error bar) and plume area (blue line). Red crosses indicate VIIRS-based IME estimates from this study, while cyan crosses show independent VIIRS estimates by de Jong et al.^16^ Shaded regions indicate distinct phases of plume evolution (I–III). (d) Three-dimensional terrain with elevation contours at 100 m intervals starting from 0 m; colored markers indicate the plume center-of-mass locations for different phases.

As shown in Figure 5a, the plume initially appears as a compact feature near the emission source and progressively disperses downwind, aligning with the prevailing wind conditions. Areas with elevated Δ*X*_CH_4__ are highlighted using a color map range of 0–3 ppm, allowing for clear visualization of concentration gradients. Throughout the observation period, the plume remains detectable in multiple frames. The emission site marked in Figure 5b, delineated by the red polygon (black cross in [Fig fig5]a due to the lower GSD), corresponds to a known compressor station, which corroborates the source of the detected plume. The spatial alignment between the methane plume and the facility strengthens the validity of MTG-FCI’s capability in pinpointing localized methane emissions.

A visual comparison of the IME of VIIRS and MTG-FCI is presented in Figure 5c. Our VIIRS-based IME estimates (red markers) are consistent with the independent results of de Jong et al.^16^ (cyan markers), while FCI-derived IME values at the VIIRS overpass times are generally lower, likely arising from the differences in spatial coverage, viewing geometry, SRF, and auxiliary inputs (e.g., masking thresholds) used during retrieval. Despite this offset, the temporal evolution and overall trends remain broadly consistent across the two sensors.

Based on the plume morphology observed in Panel (a), the IME trends shown in Panel (c), and the corresponding terrain context illustrated in Panel (d), the release event is segmented into three distinct phases: emitting (yellow, I), stabilization (red, further divided into II-a and II-b), and dispersion (blue, III). These phases are also indicated by the colored plume contours in Figure 5a for visual reference.

The first of these, Phase I (10:58–11:48 UTC), marks the initial emitting stage. During this period, IME rises steadily from 0 to approximately 300 tons, indicating sustained and active methane release. The plume remains compact and close to the source (black cross), suggesting that the emission dominates over dispersion with early signs of advection beginning to stretch it along the prevailing wind direction. This phase is dominated by local accumulation and limited downwind transport.

Phase II (11:48–14:48 UTC) represents the stabilization stage, during which the plume detaches from the source and evolves under atmospheric transport. To capture the temporal variability within this period, we further divide it into Phase II-a (11:48–12:58 UTC) and Phase II-b (12:58–14:48 UTC).

Phase II-a is a transitional phase, during which the plume detaches from the source. While IME stabilizes around 300–350 tons, the plume area increases significantly from about 100 to over 200 pixels. This decoupling between the IME and the plume area indicates that horizontal advection and turbulent mixing dominate the plume evolution, redistributing methane downwind without substantial additional emissions. Consistently, Figure 5d shows that the plume center of mass remains confined to low-elevation terrain (≤100 m) during this phase, prior to its subsequent advection across rapidly rising terrain in the following Phase II-b. The relative stability of the IME during this phase makes it the most suitable interval for estimating the total emitted mass.

In Phase II-b, both the IME and the plume area increase again despite the cessation of emissions at the source, which suggests the retrieval of artifacts. During this period, Figure 5d shows that the plume is advected northeastward across rapidly rising terrain, from elevations of ∼100 m up to ∼400 m, which could modulate the effective column mass. This effect is represented in our analysis by the fixed column height uncertainty term (σ_H_), based on a standard deviation of elevation of 350 m across the plume footprint (see details in the Supporting Information, Section S3). In addition, VIIRS-based IME estimates also show an increase during this interval, indicating that this behavior is not unique to FCI retrievals. Part of the IME increase may stem from apparent nonlocal enhancements, for example, due to water vapor-related retrieval artifacts, as also noted by de Jong et al.^16^ for VIIRS observations. As a result, Phase II-b is excluded from direct emission quantification since part of the signal likely reflects secondary effects rather than new emissions. The consistency between MTG-FCI and VIIRS-based IME estimates further supports this interpretation.

Phase III (14:48–15:58 UTC) corresponds to the dispersion stage. For the last overpasses (after 15:08), the plume mask and area used for the IME extend beyond the map area shown in Figure 5a, but they are fully included in the quantitative analysis. During this period, the IME and plume area both decline, indicating plume dilution within the boundary layer. The reduction in IME suggests that methane is no longer accumulating and is instead dispersing and mixing with the atmosphere vertically and horizontally. As the concentration approaches the detection limit, retrieval uncertainty also increases. Therefore, Phase III is also excluded from emission quantification.

The mean accumulated IME during Phase II-a is 324 ± 18 t, where the uncertainty reflects temporal variability across this phase. Individual IME retrieval errors are of similar magnitude; therefore, the phase-average is considered representative for quantification. For intersensor comparison, this value is of similar order to the VIIRS-based estimates, with our retrieval giving 310 ± 47 t (12:24 UTC) and 331 ± 40 t (12:47 UTC), while de Jong et al.^16^ reporting 344 ± 53 t. This consistency suggests that both sensors capture the magnitude of the short-term release in a comparable way despite differences in retrieval and plume masking approaches.

To estimate the average methane emission rate (*Q*, t/h), we use the average IME during Phase II-a, which follows the initial growth and precedes possible water vapor- and terrain-induced effects. Assuming a steady emission during Phase I, we calculate:

$$Q=\frac{{\overset{-}{\mathrm{IME}}}_{\mathrm{II‐a}}}{\Delta t_{I}}$$ 5

where ${\overset{-}{\mathrm{IME}}}_{\mathrm{II‐a}}$ is the mean accumulated IME during the stabilization phase (324 ± 18 tons), and Δ*t*_I_ is the duration of the initial emission phase (50 ± 10 min). The uncertainty in *Q* is computed using the standard error propagation:

$$\sigma_{Q}=Q\cdot \sqrt{{\left(\frac{\sigma_{\mathrm{IME}}}{\overset{-}{\mathrm{IME}}}\right)}^{2}+{\left(\frac{\sigma_{\Delta t}}{\Delta t}\right)}^{2}}$$ 6

Applying this to our case yields an estimated averaged methane emission rate of *Q* = 389 ± 81 t/h lasting for ∼50 min, indicating a significant short-term release of methane into the atmosphere.

### 3.3 Implications and Future Directions

Through an integrated evaluation involving end-to-end simulations, regional detection limit analysis, and a real transient emission event, we provide a comprehensive assessment of MTG-FCI’s ability in methane monitoring. The results presented in this study demonstrate that the MTG-FCI can detect, quantify, and track methane plumes under realistic conditions. Building on these findings, this section discusses the broader implications for geostationary-based monitoring and outlines potential directions for future development.

Simulation experiments using WRF-LES plume fields indicate that the current MBMP retrieval framework can detect emissions above 30–50 t/h under typical Algerian conditions. This detection range represents a realistic lower bound for local applications, given the spatial resolution and signal dilution associated with MTG-FCI’s pixel footprint. At the regional scale, the minimum detectable emission rate reaches ∼20–30 t/h across central disk regions with favorable viewing geometry and bright homogeneous surfaces, representing the best detection sensitivity achievable by the MTG-FCI. However, regions with heterogeneous surfaces, oblique viewing angles, or persistent cloud cover exhibit higher *Q*_min_ values or apparent retrieval gaps.

We also demonstrate the MTG-FCI’s real-world performance using data from September 29, 2023, when a transient methane release from a compressor station in Algeria was successfully detected and dynamically tracked. The full lifecycle of the plume from onset to dispersion was successfully captured at high temporal resolution. The emission rate, estimated at 389 ± 81 t/h, was independently validated by VIIRS observations from Suomi-NPP, JPSS-1, and JPSS-2 from this study and independent research,^16^ confirming the reliability of both the MBMP algorithm and the IME-based quantification. Continuous 10 min sampling enabled phase-resolved analysis of plume dynamics, distinguishing between emission-driven growth and transport-dominated dispersion. For this short-lived release, the total IME (∼300 t) also provides useful but distinct information, which is particularly relevant for assessing the environmental impact of transient events, highlighting a practical advantage of geostationary sensors, where continuous sampling constrains both the onset and cessation of emissions and enables event-scale emission estimation for transient releases.

Beyond this case, additional events further illustrate the lower detection bound and surface-dependence of MTG-FCI retrievals (see Supporting Information, Section S4). A subsequent release from the same Algerian facility on October 1, 2023 (∼40–50 t,^16^) was clearly detectable in its early emitting phase, where the per-frame IME reached ∼65 t (Supporting Information, Section S4.1, Figure S6). In later frames, plume delineation relied almost entirely on spatiotemporal coherence and manual mask refinement, showing that plumes with a per-frame IME of ∼30–50 t are only marginally detectable and unsuitable for robust quantification. In contrast, a plume reported in Russia on March 19, 2025 (listed in the IMEO hotspot database) with an emission rate of 191 ± 63 t/h according to SRON’s TROPOMI-based weekly report,^39^ was only faintly visible in the MTG-FCI retrievals and could not be confidently extracted due to dark, heterogeneous surface conditions and the absence of coincident FCI observations during the reported hour (Supporting Information, Section S4.2, Figure S7). In this case, the local *Q*_min_ (∼224 t/h) even exceeded the reported emission rate, underscoring that detectability is governed less by *Q* values alone and more by the combined strength of per-pixel enhancements and plume spatial extent. As a result, for MTG-FCI, even very large reported emissions may remain undetectable under adverse conditions, whereas more moderate releases can sometimes still be traced if the per-frame IME is sufficiently strong.

This contrast between emission rates *Q* and the directly observable IME emphasizes an important methodological consideration. Frameworks for limits of detection, such as our end-to-end simulation and *Q*_min_ estimation, inherently assume continuous and steady emissions during the observation period. This is a reasonable approximation for polar-orbiting instruments within single overpasses but not fully representative for short-lived, transient releases. While *Q* is essential for characterizing emission dynamics, the IME provides a direct measure of the total atmospheric burden, which would be more relevant for transient events. In this context, the key advantage of geostationary observations lies in their continuous temporal sampling, which allows the full temporal evolution of a release event to be resolved rather than inferred from isolated snapshots. Besides this, an additional methodological advantage of geostationary observations lies in their strong spatiotemporal coherence. The continuous temporal sampling of the MTG-FCI allows methane plumes to be identified not only from instantaneous spatial patterns but also from their coherent advection and persistence in time. This provides a powerful constraint for automated detection, helping to suppress false positives arising from clouds, surface heterogeneity, and random noise. Future developments may exploit this property through time-series-based detection frameworks or physics-informed machine learning approaches that explicitly encode plume continuity and transport dynamics.

Placed in a broader monitoring context, this methodological advantage highlights the complementary role of geostationary missions within the global satellite constellation. The MTG-FCI joins sensors such as GOES-ABI, which provides similar temporal capabilities but is limited to the Americas, and Himawari-8, which covers the Asia-Pacific region. Together, these platforms establish the foundation for a global, high-frequency methane monitoring network capable of resolving short-lived or intermittent releases that may be missed by polar-orbiting sensors with lower revisit frequency. While geostationary instruments are generally sensitive only to relatively large emission events, these significant releases often dominate regional and global methane budgets, making their timely detection and attribution especially critical for mitigation efforts.

In summary, the MTG-FCI serves as an important complement to the global methane monitoring constellation. Its high-temporal coverage, optimized SWIR bands, and subkilometer resolution can, in principle, enable the detection and quantification of large emission events under favorable conditions. The Algerian compressor station release on September 29, 2023 (total mass ∼300 tons), where the full plume lifecycle was captured and independently validated against VIIRS, demonstrates the instrument’s capability for a robust quantification of major emissions. In contrast, the March 2025 Russia case (∼191 ± 63 t/h reported by the TROPOMI detected list) illustrates the limitations of retrievals over dark, heterogeneous surfaces, where detectability is hampered despite the large reported emission rate. Together, these contrasting examples underscore that the MTG-FCI is best suited for synergy with higher-sensitivity polar-orbiting missions, which provide a broader spectral information and initial plume identification. This complementary role is particularly valuable for near-real-time alert systems, verification protocols, and targeted field response.

Future improvements should focus on (i) reducing retrieval noise through adaptive filtering and developing robust automated detection algorithms, (ii) refining detection metrics beyond *Q*, for example, by explicitly incorporating an instantaneous IME as a complementary indicator of detectability for geostationary observations, and (iii) leveraging the spatiotemporal continuity of GEO data to mitigate retrieval challenges over complex and heterogeneous surfaces. Such advances, combined with synergistic analysis across GEO and polar sensors, will strengthen the completeness of methane monitoring and support science-based policy interventions such as independent verification of reported emissions and prioritization of mitigation actions at major facilities.

## Supplementary Material





## Notes

Animation S1 (PDF)
Details of coregistration and displacement analysis, including interband/date/time misalignment statistics; cloud screening approach and uncertainty assessment; figures and tables showing regional detection performance; two supplementary methane plume cases (Algeria, Oct 1, 2023; Russia, Mar 19, 2025) with retrieval maps and discussion; and an appendix with extended methodological notes (PDF)
