# Supplementary figures and images for: Assessing the Potential of the MTG-FCI Geostationary Mission for the Detection of Methane Plumes
Source: Environ Sci Technol. 2026 Feb 16;60(8):6137–48. doi: 10.1021/acs.est.5c07974 (PMC12961945; doi:10.1021/acs.est.5c07974)

08:00 UTC

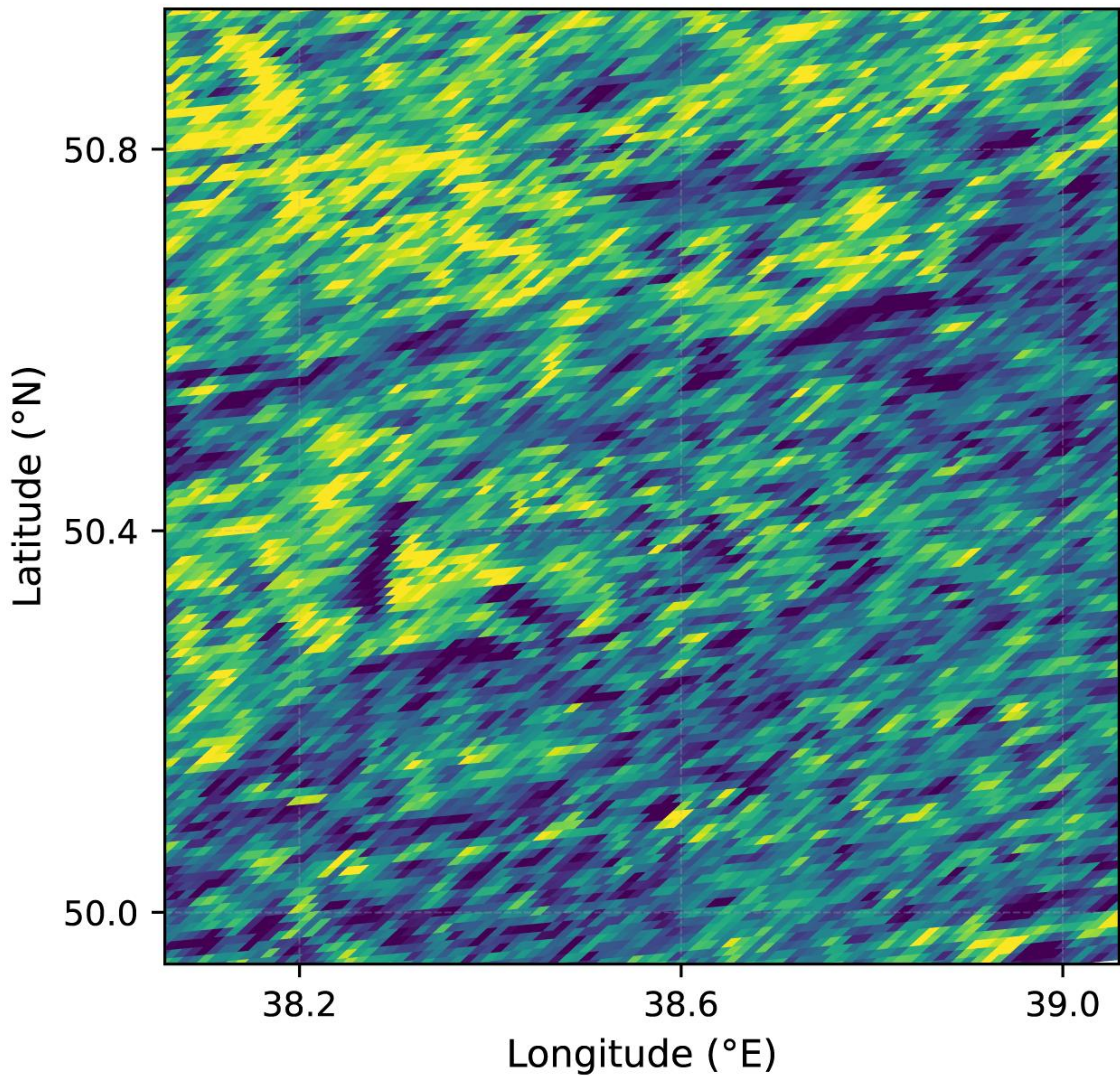

08:10 UTC

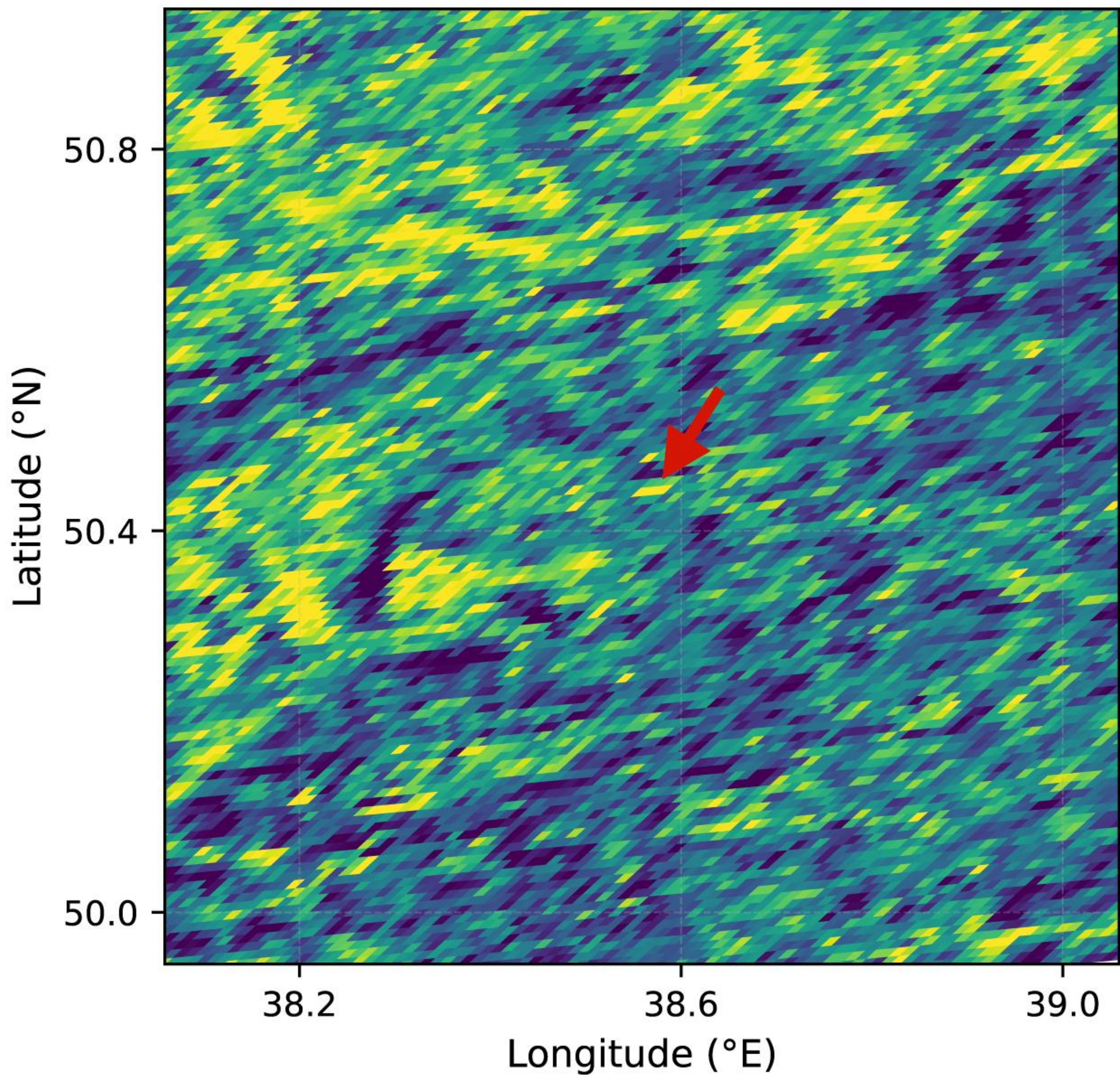

08:20 UTC

Latitude ( $^{\circ}$ N)

50.8

50.4

50.0

38.2

38.6

39.0

Longitude ( $^{\circ}$ E)

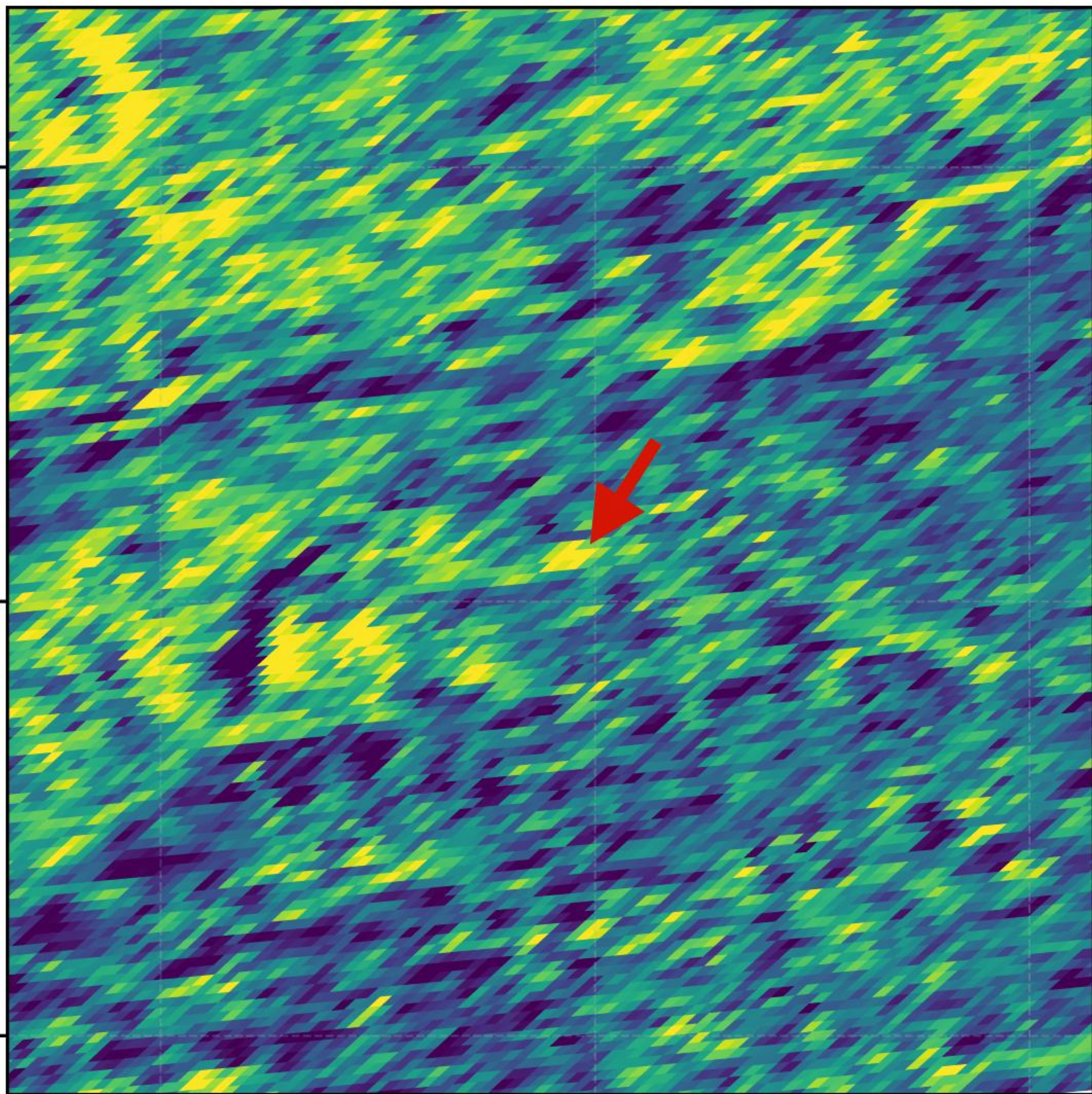

08:30 UTC

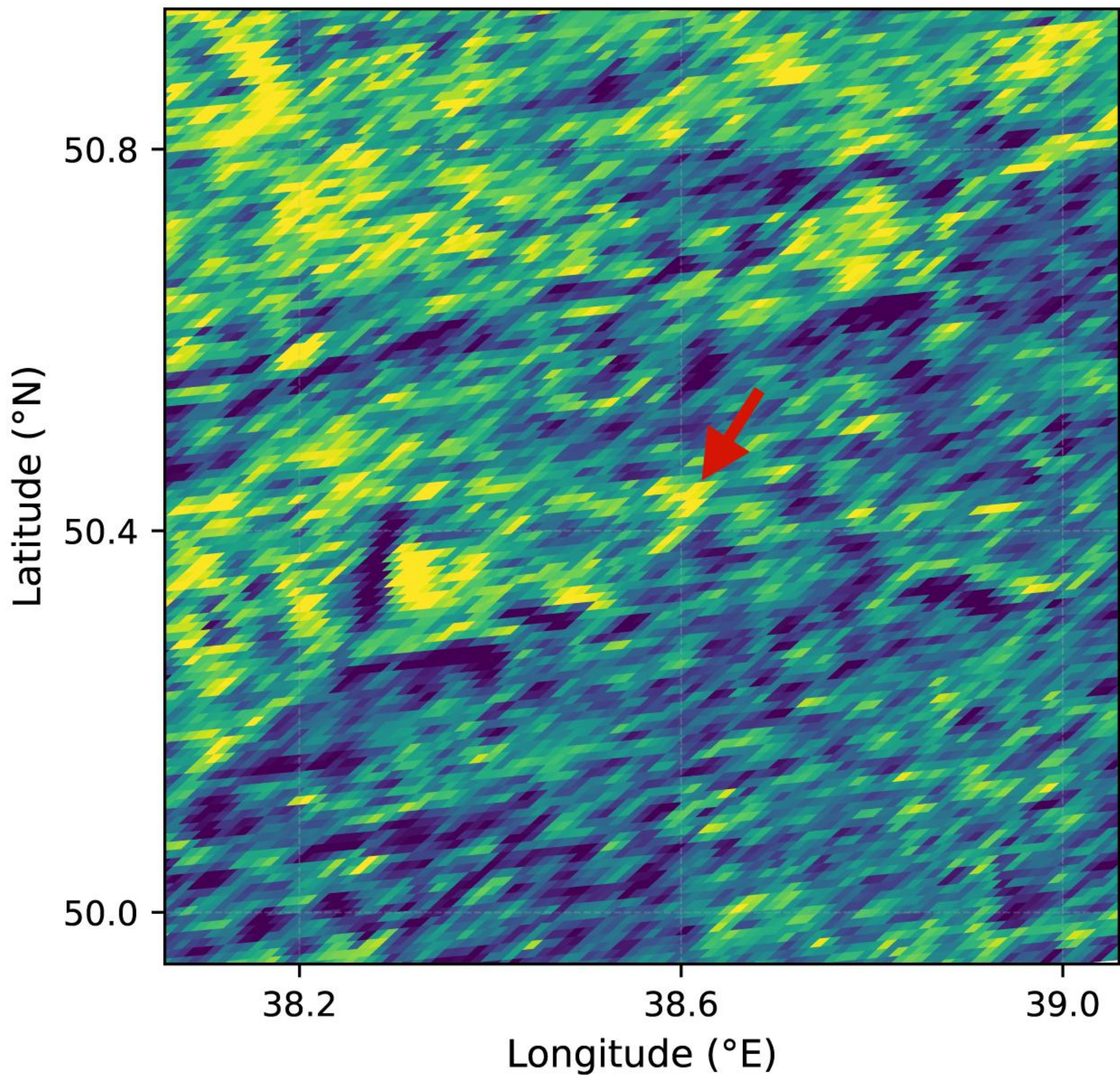

08:40 UTC

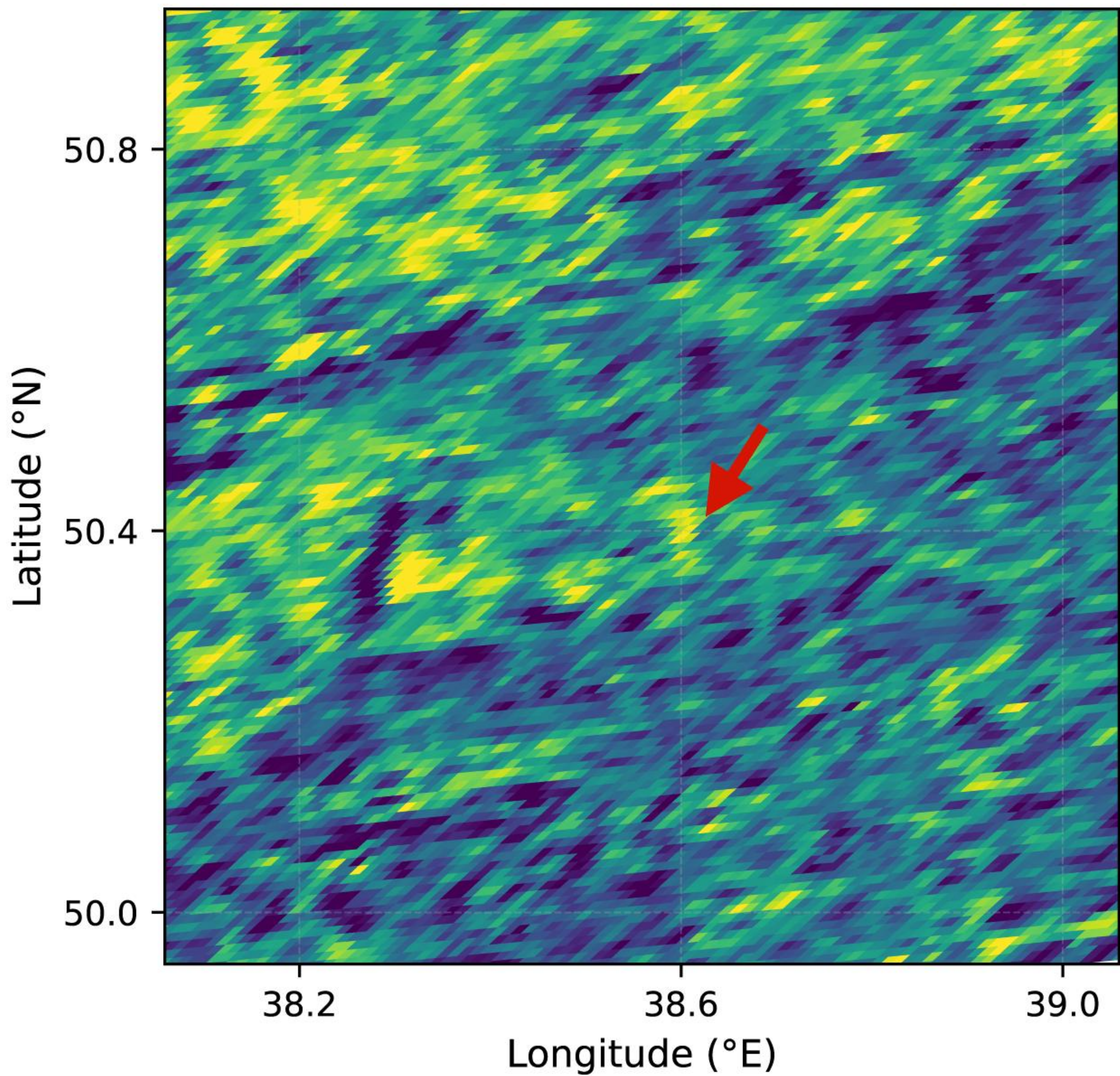

08:50 UTC

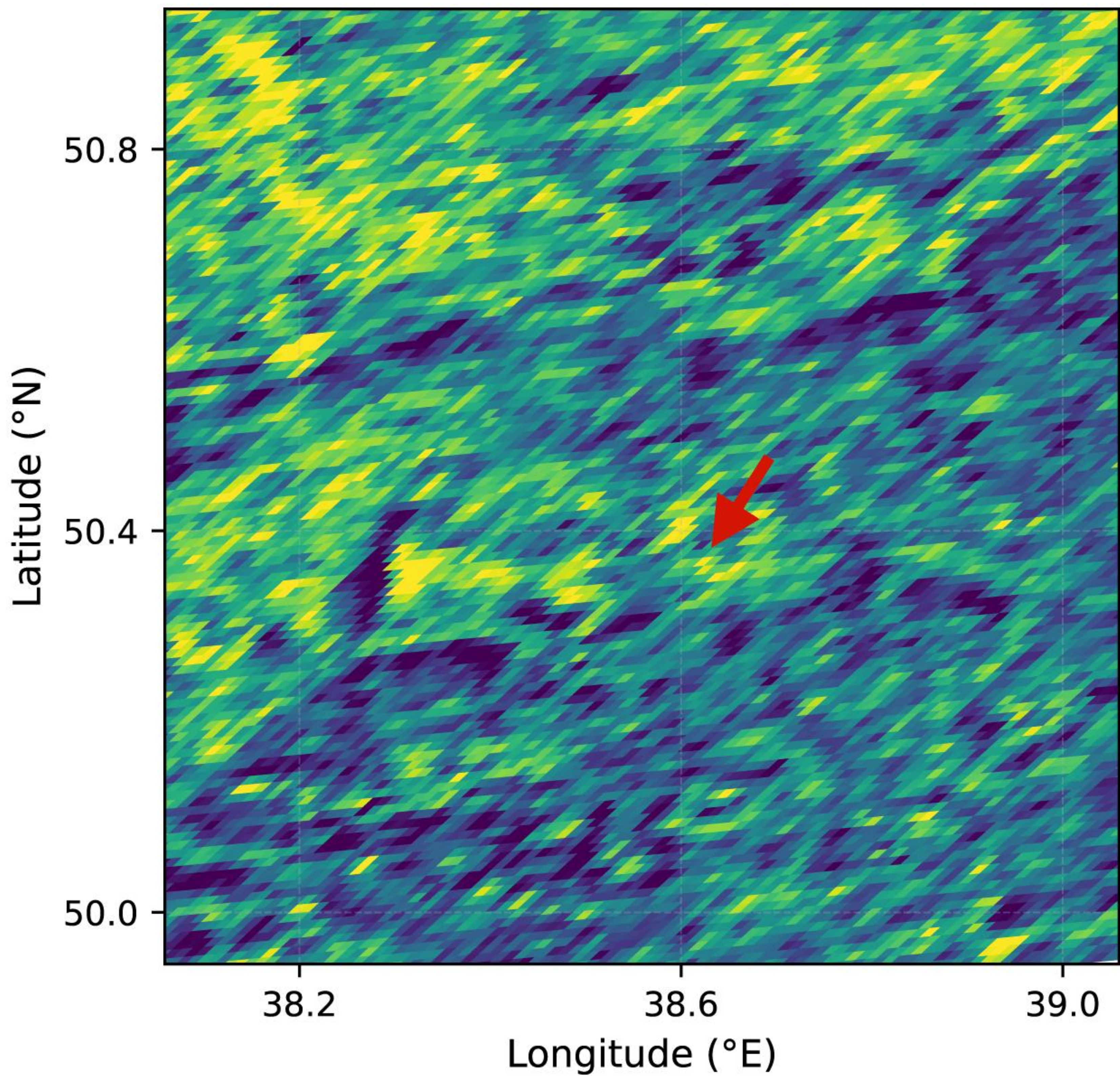

09:00 UTC

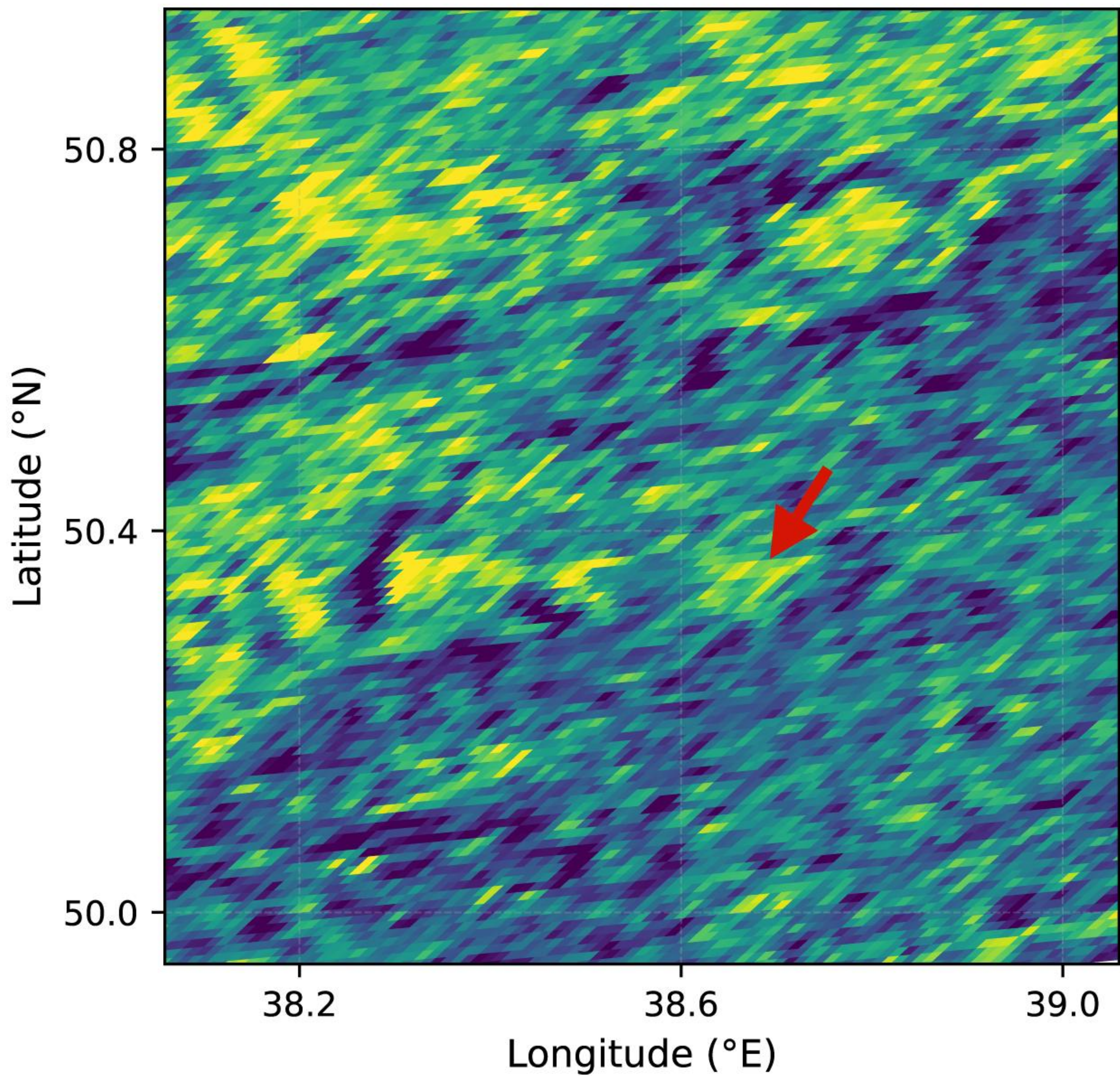

09:10 UTC

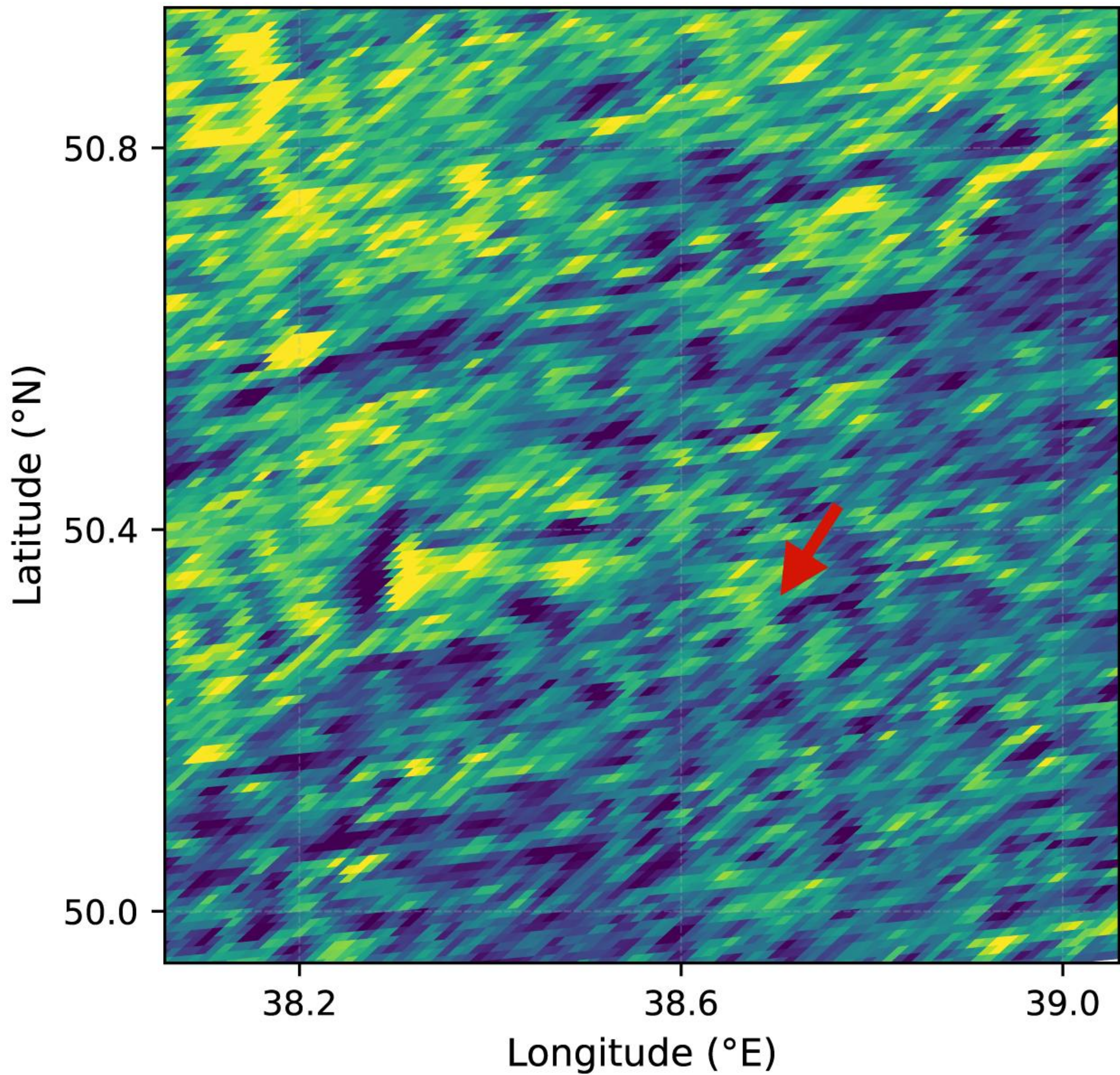

09:20 UTC

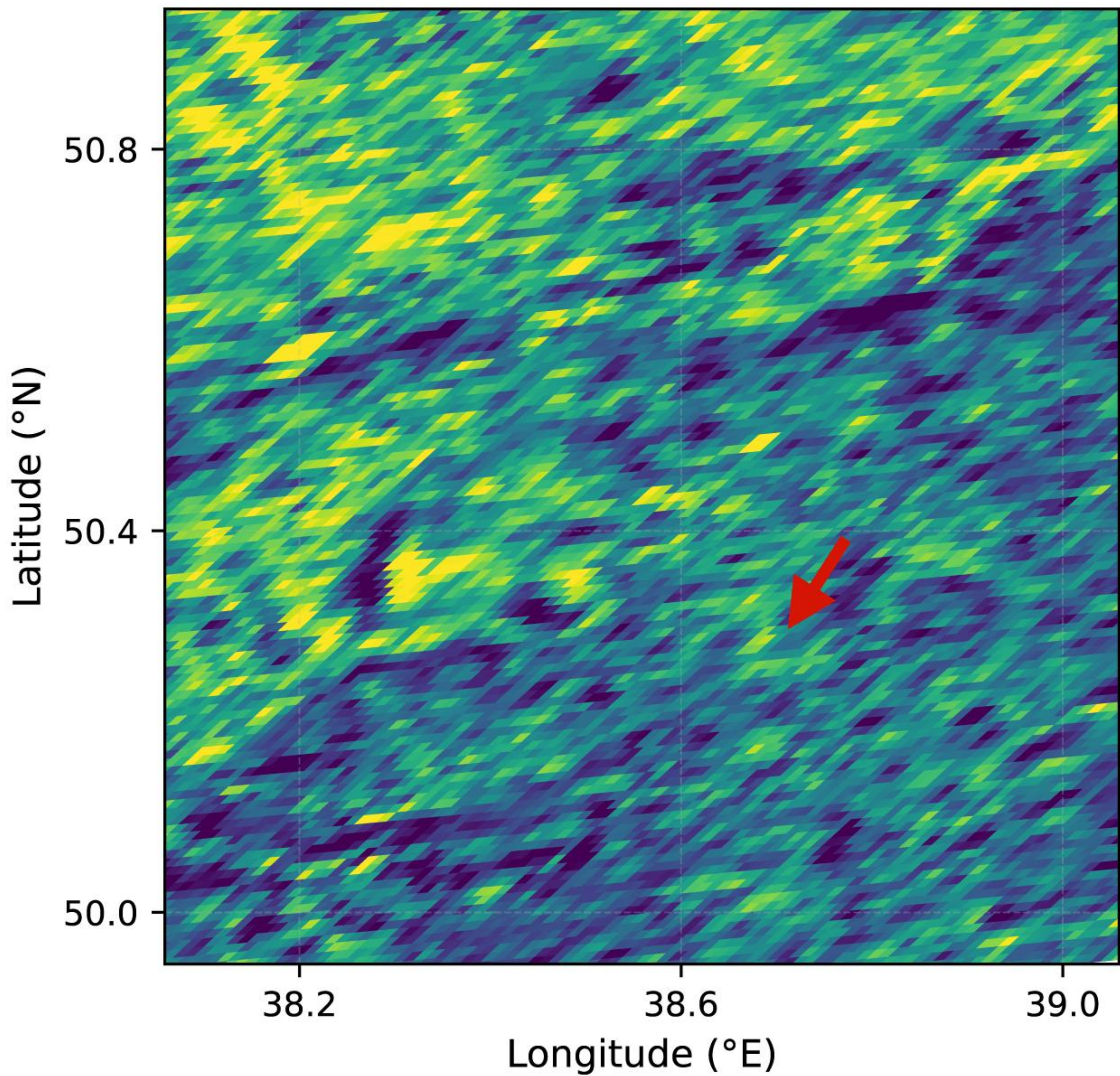

09:30 UTC

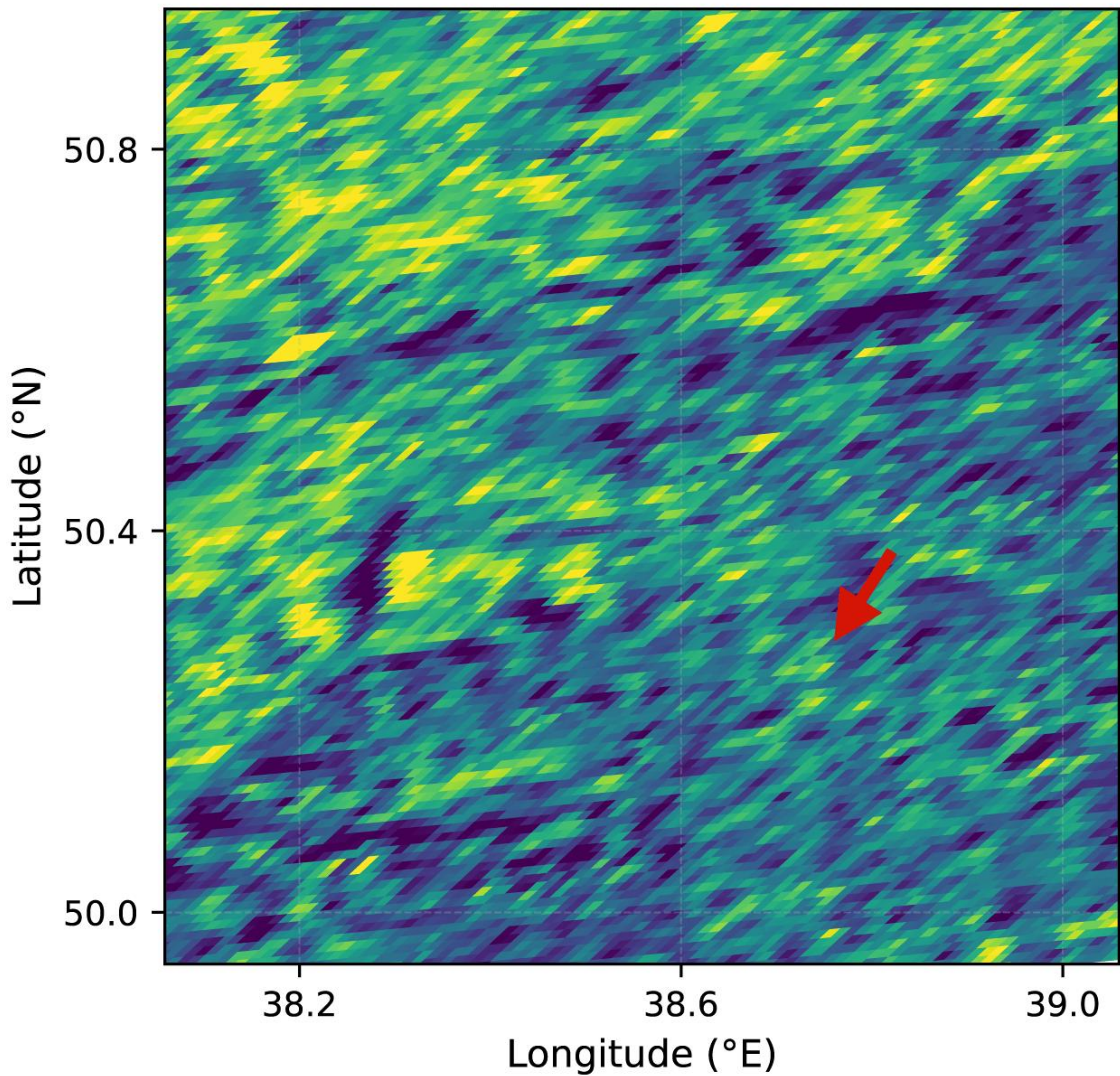

09:40 UTC

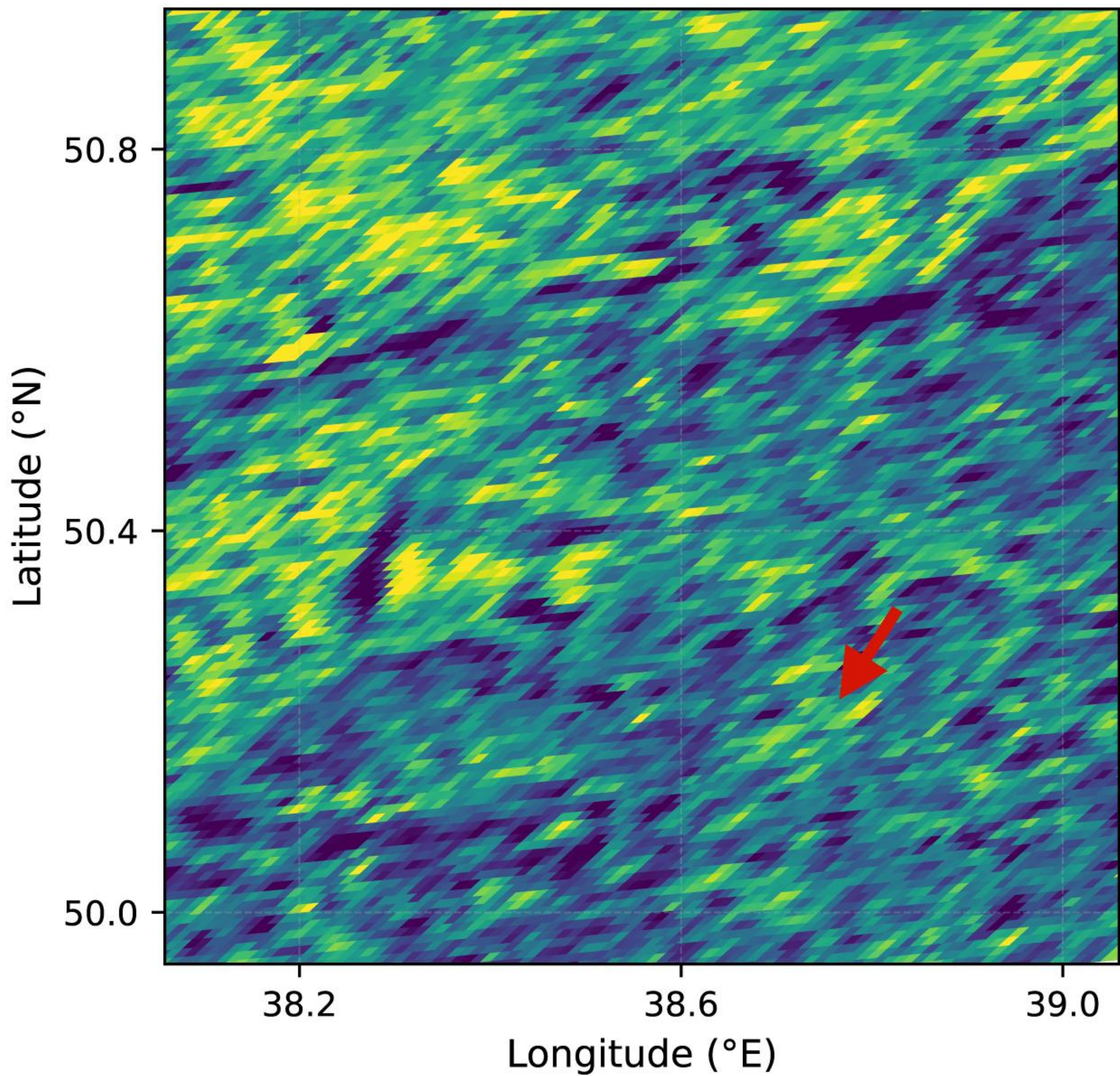

09:50 UTC

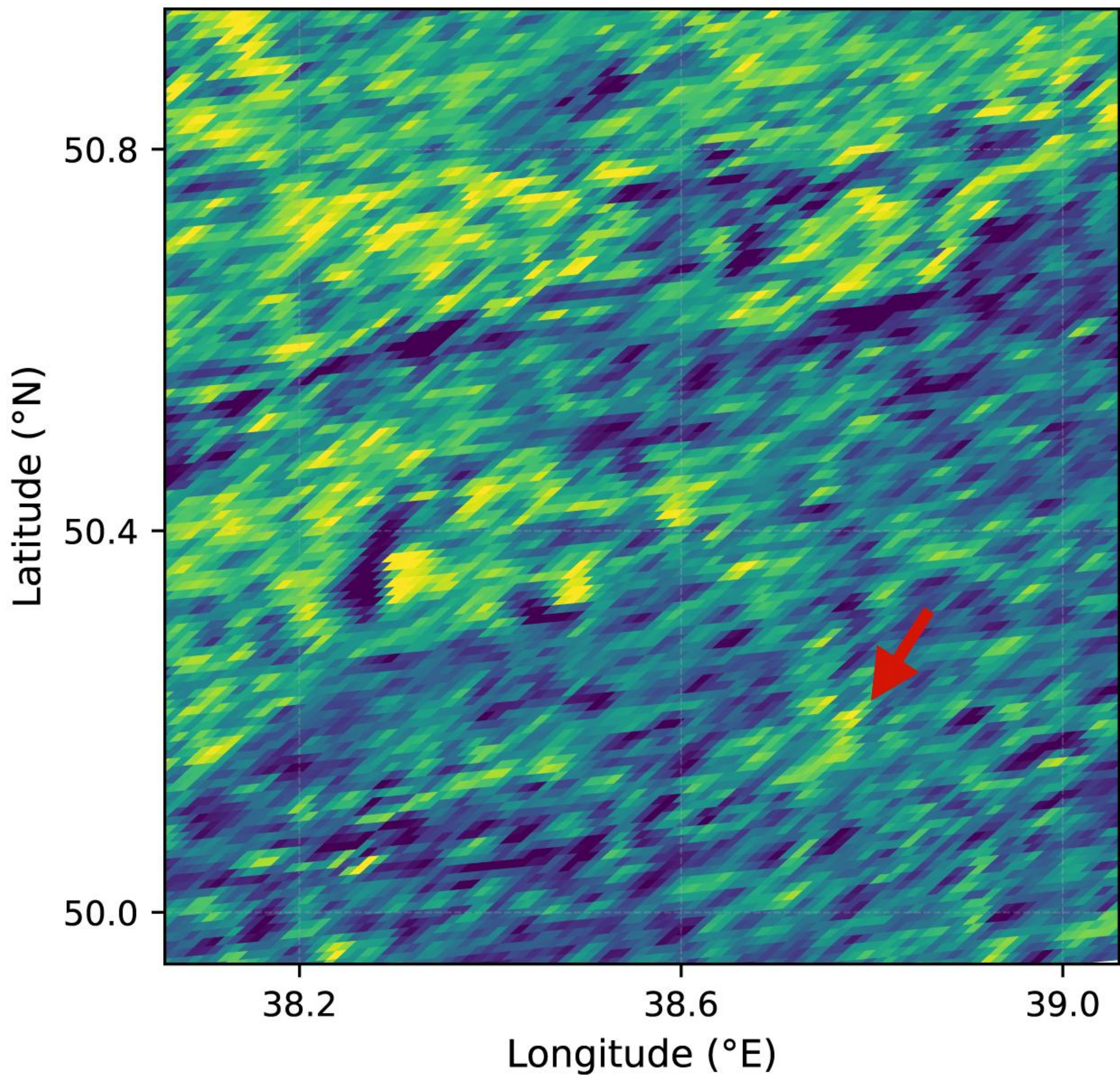

Supplement: Supplementary file 1 [file es5c07974_si_001.pdf]
