# Supplementary material for: Assessing the Potential of the MTG-FCI Geostationary Mission for the Detection of Methane Plumes
Source: Environ Sci Technol. 2026 Feb 16;60(8):6137–48. doi: 10.1021/acs.est.5c07974 (PMC12961945; doi:10.1021/acs.est.5c07974)
Supplement: Supplementary file 2 [file es5c07974_si_002.pdf]

Supporting Information for

Assessing the potential of the MTG-FCI geostationary mission for the detection of methane plumes

Shanyu Zhou[a,*], Javier Gorroño[a], Javier Roger[a], Itziar Irakulis-Loitxate[a,b], Rasmus Lindstrot[c], Zhipeng Pei[d], Lulu Si[a], Luis Guanter[a,e]

[a] *Research Institute of Water and Environmental Engineering (IIAMA), Universitat Politècnica de València, Valencia, 46022, Spain*

[b] *International Methane Emissions Observatory, United Nations Environment Programme, Paris, 75015, France*

[c] *EUMETSAT, Darmstadt, 64295, Germany*

[d] *State Key Laboratory of Information Engineering in Surveying, Mapping and Remote Sensing, Wuhan University, Wuhan, 430072, China*

[e] *Environmental Defense Fund, Amsterdam, 1083 HN, The Netherlands*

*Corresponding author: `szhou1@upv.es`

Contents:

This Supporting Information includes:

Tables S1–S3. Displacement statistics for inter-band, inter-date, and inter-time registration.
Tables S4. MTG-FCI acquisition dates used for $Q_{\min}$ estimation.
Figures S1–S3. Maps and histograms of displacement fields for Algeria and Iran.
Figure S4. Effect of co-registration on methane plume retrievals.
Figure S5. Time series of IME with error decomposition.
Figure S6. Retrieval of the 1 October 2023 Algerian case
Figure S7. Retrieval of the 19 March 2025 Russian case
Animation S1. Animated retrieval of the 19 March 2025 Russian case

Number of pages: 15
Number of tables: 4
Number of figures: 7

S1 Co-registration and Displacement Analysis

Accurate methane retrieval with MTG-FCI requires precise spatial alignment both across spectral bands and across repeated observations. Small misalignments propagate into the band ratios used by MBMP and can introduce plume artifacts and biases. Following the geometric-accuracy terminology used for geostationary imagers, we treat these as cases of *relative geometric accuracy* (RGA): band-to-band, day-to-day, and scan-to-scan registration, and quantify them with displacement statistics rather than external ground truth (Aksakal, 2013). Currently, no dedicated and peer-reviewed assessment of MTG-FCI geometric accuracy is available; therefore, the analysis presented here is restricted to relative displacement diagnostics in the context of methane retrieval.

These three displacement categories have distinct implications for methane monitoring:

- **Inter-band (NIR1.6 vs. NIR2.2):** directly affects single-scene retrievals, since MBMP relies on ratios between NIR1.6 and NIR2.2 radiances; even sub-pixel offsets can distort plume morphology.
- **Inter-date (consecutive days):** impacts retrievals that subtract a reference day to compute enhancements; residual shifts may cause spurious $\Delta X_{\mathrm{CH_4}}$ signals over heterogeneous surfaces.
- **Inter-time (10-min scans):** does not bias individual retrievals but affects the temporal consistency of Integrated Methane Enhancements (IME) and subsequent emission-rate (Q) estimates, since systematic drifts can alter the apparent plume motion within the FCI observing cycle.

To quantify these effects, we employed the GeFolki optical-flow algorithm (Aplyer) to estimate dense pixel-wise displacement fields between reference and target images, and converted the results into metric units using the local ground sampling distance of the NIR2.2 channel. Detailed methodology and statistics are reported below.

S1.1 Methodology

Reference band and resampling For all co-registration tasks, the NIR2.2 channel acquired at the target date and time was selected as the spatial reference grid, since it provides the highest spatial resolution among the near-infrared channels (0.5 km at nadir). The NIR1.6 channel, which has a native resolution of 1 km, was first geometrically resampled onto the NIR2.2 grid prior to displacement estimation. We used bilinear interpolation to upsample NIR1.6, as it preserves spatial gradients and radiometric consistency while avoiding the blocky artifacts introduced by nearest-neighbor resampling.

An alternative approach would be to downsample the NIR2.2 channel to match the coarser 1 km NIR1.6 grid. However, this would discard half of the available spatial information and lead to unnecessary smoothing of methane plume structures, which are often narrow and close to the detection limit. Moreover, the 2.2 μm band coincides with the strongest absorption features of methane accessible to FCI, and therefore serves as the primary channel for methane retrievals in the MBMP framework. Preserving its native spatial resolution is essential to maximize sensitivity to plume morphology and to avoid dilution of the absorption signal.

Although upsampling does not add new information to NIR1.6, it provides consistent pixel-to-pixel correspondence across bands, which is a prerequisite for accurate displacement estimation and ratio-based methane retrievals. In this way, the co-registration strategy prioritizes both the spatial fidelity and the spectral sensitivity required for robust methane detection.

Alignment and displacement estimation We employed the GeFolki optical-flow method (Aplyer; Plyer et al., 2015; Brigot et al., 2016) to perform spatial co-registration of spectral data and to estimate dense pixel-wise displacement fields. Given a reference and a moving image, GeFolki returns the displacement components (u, v) on the reference grid, which describe the shifts required to align the two images. The estimated flow is subsequently applied to the moving image using `wrapData` to complete the registration. By convention, positive u points toward increasing column indices and positive v toward increasing row indices in image space, avoiding ambiguity with geographic directions.

To systematically assess co-registration performance, we considered three displacement categories: (i) inter-band (NIR1.6 vs. NIR2.2 at the same time), (ii) inter-date (NIR2.2 on consecutive days at the same scan time), and (iii) inter-time (successive NIR2.2 scans within the same day relative to 11:48 UTC). These cover band-to-band alignment, day-to-day repeatability, and short-term pointing stability, respectively.

All displacements are first computed in pixels (u, v). To convert them into metric units, we derive the local ground sampling distance (GSD) from the NIR2.2 lon–lat grid. For each pixel (i, j) with longitude $\lambda_{i,j}$ and latitude $\varphi_{i,j}$, the approximate longitudinal and latitudinal distances are given by

$$\Delta x_{i,j} \approx \Delta\lambda_{i,j} \cdot R \cos\varphi_{i,j}, \qquad \Delta y_{i,j} \approx \Delta\varphi_{i,j} \cdot R, \tag{1}$$

where $R \approx 6371\,\text{km}$ is the Earth's mean radius, and $\Delta\lambda_{i,j}, \Delta\varphi_{i,j}$ are the longitude and latitude differences to adjacent pixel centers (in radians). The corresponding ground sampling distances in meters are

$$dx_m = |\Delta x_{i,j}| \times 1000, \qquad dy_m = |\Delta y_{i,j}| \times 1000. \tag{2}$$

Pixel displacements are then converted as

$$u_m = u \cdot dx_m, \qquad v_m = v \cdot dy_m, \qquad |d| = \sqrt{u_m^2 + v_m^2}. \tag{3}$$

This ensures that reported displacements reflect both pixel-scale variability and the effective ground resolution at the scene.

Regions and dates Two 2° × 2° ROIs were analyzed: Algeria (35.0°N, 6.5°E, near disk center) and Iran (33.0°N, 57.0°E, closer to disk edge). Clear-sky pairs were selected from commissioning data (30 Sep–1 Oct 2023) and publicly released data (14–15 Jun 2025). These datasets are independent of the plume case (29 Sep 2023, Algeria) and serve as diagnostics of instrument- and geometry-driven displacements.

S1.2 Displacement statistics and visualization

Residual misalignments were quantified across three categories: inter-band, inter-date, and inter-time. For each case, we report mean ± std of $|d|$ in pixels and meters (Tables S1–S3). Representative examples are shown in Figures S1–S3 for Algeria (top) and Iran (bottom), including reference and target images, displacement fields, and histograms of $(u, v, |d|)$.

Inter-band displacements. Offsets between NIR1.6 and NIR2.2 are consistently on the order of ∼0.6 px, but the absolute distances differ between regions (Table S1, Fig. S1). In Algeria, located near disk center, this corresponds to ∼340 m, while in Iran, closer to the disk edge, the same pixel offset translates to ∼700 m.

Such inter-band residuals are on the order of 0.5–1 px, which is comparable in magnitude to the relative geometric accuracy reported for SEVIRI Aksakal (2013). However, for MTG-FCI, these offsets cannot be uniquely attributed to instrument geometry alone. They likely result from a combination of factors, including spatial resampling of the NIR1.6 channel, surface-induced spectral contrast differences between bands, and viewing-geometry effects, particularly toward the disk edge where the ground-projected displacement becomes larger. For this reason, systematic co-registration is applied as an operational alignment step to ensure sub-pixel consistency for methane retrieval.

Table S1: Inter-band displacement statistics between NIR1.6 and NIR2.2. Reported values are mean ± std of $|d|$ in pixels and meters.

Region	Date	Time	$\|d\|$ (px)	$\|d\|$ (m)
Algeria	20231001	11:58	0.58 ± 0.37	341 ± 215
		12:08	0.58 ± 0.37	343 ± 215
		12:18	0.57 ± 0.37	339 ± 217
Iran	20250614	11:58	0.60 ± 0.31	723 ± 415
		12:08	0.60 ± 0.30	716 ± 398
		12:18	0.60 ± 0.30	725 ± 399

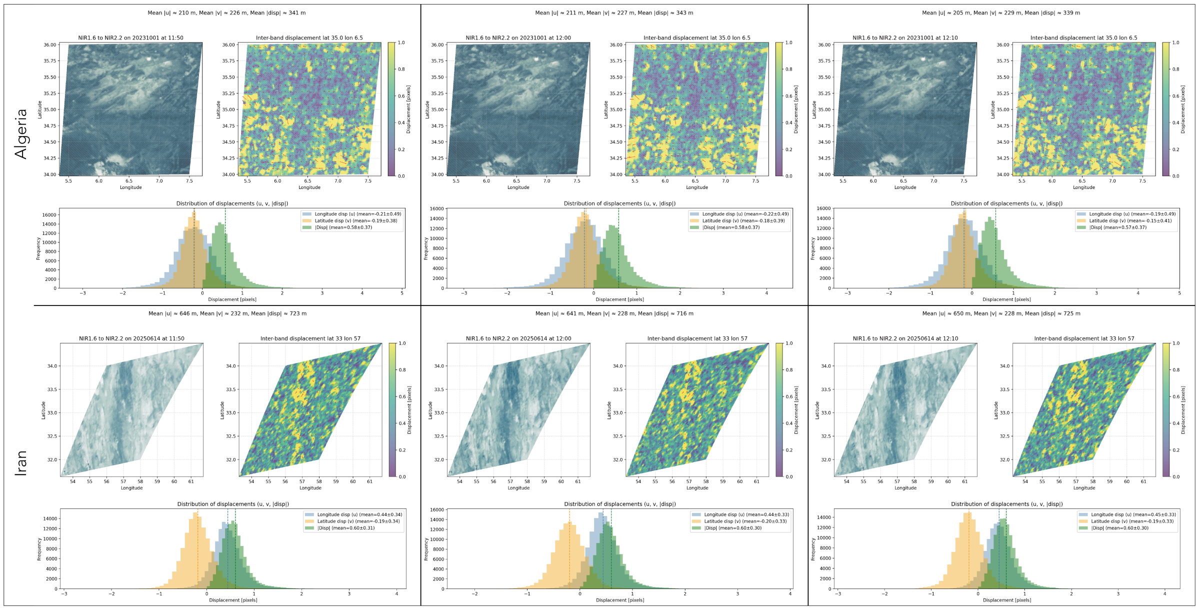

Figure S1: Inter-band displacements between NIR1.6 and NIR2.2 channels. Systematic offsets of 200–700 m are observed across different regions. Algeria (top) and Iran (bottom).

Inter-date displacements Day-to-day displacements are slightly smaller (∼0.3–0.5 px) but show stronger variability in orientation, particularly in the Iran ROI near the disk edge (Table S2, Fig. S2). This variability likely reflects a combination of effects, including differences in viewing geometry, residual pointing uncertainties, and scene-dependent radiometric changes driven by varying solar illumination and surface bidirectional reflectance (BRDF) effects between consecutive days. These effects become more pronounced at larger viewing zenith angles near the disk edge, where parallax and projection effects further amplify apparent displacements. Similar inter-day variability has been documented for SEVIRI and AHI (Debaecker et al., 2021; Yamamoto et al., 2020). Although the observed magnitudes remain within the nominal geolocation accuracy, they infer the need for systematic co-registration when applying retrieval algorithms sensitive to sub-pixel misalignments, such as the band-ratio–based MBMP approach.

Table S2: Inter-date displacement statistics from the NIR2.2 band, comparing consecutive days (1 Oct vs. 30 Sep 2023 for Algeria; 14 vs. 15 Jun 2025 for Iran).

Region	Date Pair	Time	$\|d\|$ (px)	$\|d\|$ (m)
Algeria	20231001/20230930	11:58	0.33 ± 0.15	187 ± 83
		12:08	0.36 ± 0.19	198 ± 99
		12:18	0.54 ± 0.13	350 ± 82
Iran	20250614/20250615	11:58	0.55 ± 0.15	658 ± 199
		12:08	0.49 ± 0.14	447 ± 134
		12:18	0.45 ± 0.18	439 ± 180

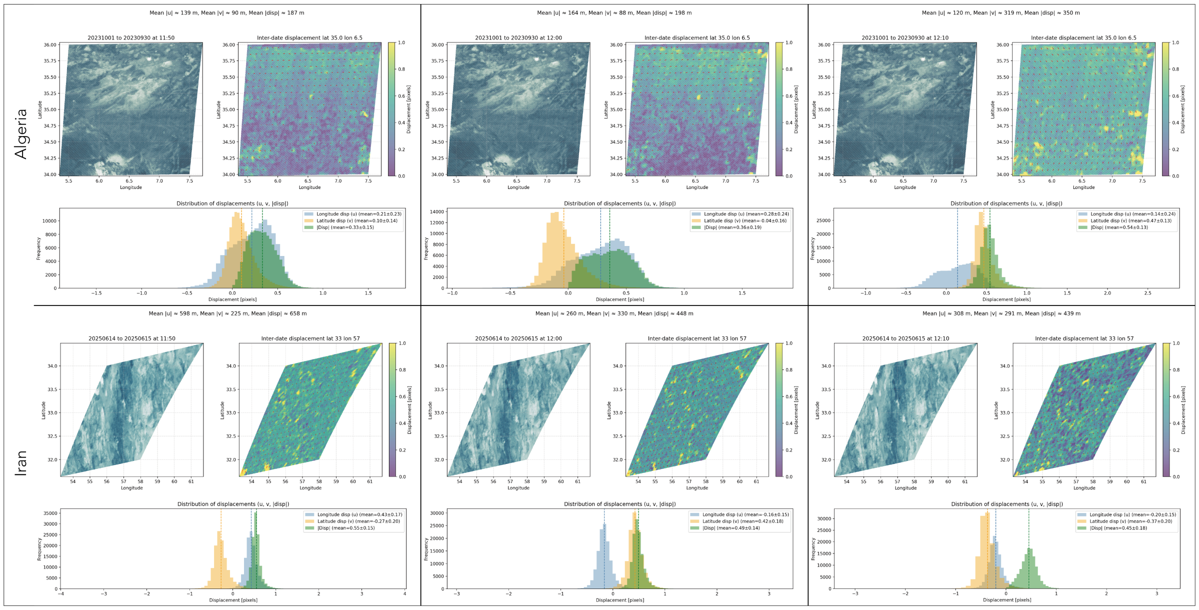

Figure S2: Inter-date displacements of MTG-FCI imagery over Algeria (top) and Iran (bottom).

Inter-time displacements Temporal displacements are the most variable among the three categories, ranging from 0.2 to 1.2 px (128–666 m in Algeria; 296–674 m in Iran. Table S3, Fig. S3).

In both regions, the apparent inter-time displacements often exhibit a coherent direction across the full scene. This behavior likely results from a combination of residual pointing variations, scan-geometry effects, and scene-dependent radiometric differences associated with changing solar illumination conditions. These effects jointly contribute to the observed apparent displacements, particularly under oblique viewing geometries near the disk edge. Such systematic scene-wide shifts do not bias individual single-scan MBMP retrievals, but they directly affect the temporal consistency of plume tracking, IME evolution, and consequently the derived emission rates (Q). This highlights the necessity of enforcing consistent inter-scan co-registration for time-resolved emission analysis.

In Algeria, located near disk center, the temporal displacements fluctuate more strongly between scans, whereas in Iran, closer to the disk edge, the magnitudes are slightly larger on average, reflecting stronger projection effects.

Coherent scene-wide drifts can alter plume masks and apparent plume motion between successive scans. To ensure temporal consistency, all scans are co-registered to a fixed reference time. These observed drifts are in line with residual navigation and scan-geometry effects reported for other geostationary imagers Aksakal (2013); Debaecker et al. (2021).

Table S3: Inter-time displacement statistics relative to the 11:40 reference scan, for Algeria (35.0°N, 6.5°E, 1 Oct 2023) and Iran (33.0°N, 57.0°E, 15 Jun 2025). Reported values are mean ± std of $|d|$ in pixels and meters.

Region	Date	Time	$\|d\|$ (px)	$\|d\|$ (m)
Algeria	20231001	11:28	0.22 ± 0.14	128 ± 85
	ref. 11:48	11:38	0.78 ± 0.13	413 ± 69
		11:58	0.65 ± 0.13	348 ± 68
		12:08	0.97 ± 0.16	613 ± 104
		12:18	0.56 ± 0.17	367 ± 109
		12:28	0.26 ± 0.22	159 ± 122
		12:38	1.17 ± 0.29	666 ± 159
		12:48	0.82 ± 0.31	495 ± 169
		12:58	0.76 ± 0.34	464 ± 188
		13:08	0.47 ± 0.36	270 ± 198
Iran	20250614	11:28	0.48 ± 0.17	497 ± 192
	ref. 11:48	11:38	0.52 ± 0.18	529 ± 198
		11:58	0.51 ± 0.11	626 ± 166
		12:08	0.43 ± 0.18	543 ± 232
		12:18	0.29 ± 0.18	296 ± 198
		12:28	0.36 ± 0.15	318 ± 144
		12:38	0.37 ± 0.17	339 ± 168
		12:48	0.68 ± 0.12	674 ± 139
		12:58	0.61 ± 0.17	500 ± 147
		13:08	0.54 ± 0.13	639 ± 196

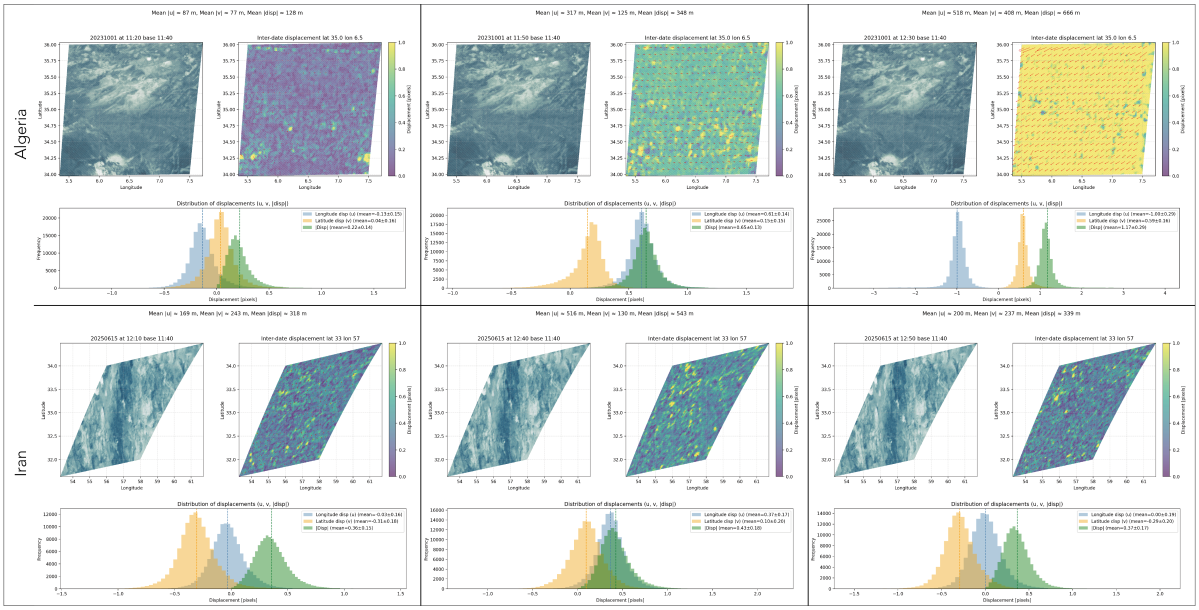

Figure S3: Inter-time displacements relative to the 11:40 reference. For each region (Algeria, top; Iran, bottom), three representative cases are shown: the minimum, median, and maximum observed displacement within the sequence (see Table S3 for full statistics across all ten scans).

Summary Residual displacements in MTG-FCI are consistently below one pixel and thus remain within the nominal relative geometric accuracy, but they differ in magnitude and character across displacement categories. Inter-band offsets are typically on the order of ∼0.6 px, corresponding to ∼340 m near the disk center (Algeria) and up to ∼700 m toward the disk edge (Iran), reflecting a combination of instrumental sampling differences and viewing-geometry–related projection effects. Inter-date shifts are slightly smaller (∼0.3–0.5 px) but more variable in direction, especially for the Iran ROI, indicating reduced spatial repeatability under oblique viewing and varying illumination conditions. By contrast, inter-time displacements within the 10-minute scan cycle are the most variable (0.2–1.2 px) and often exhibit coherent scene-wide patterns, likely arising from a combination of residual pointing variations, scan geometry, and scene-dependent radiometric effects.

While these residuals do not bias individual single-scan MBMP retrievals, they directly affect the temporal consistency of plume tracking, IME evolution, and consequently the derived emission rates. Systematic co-registration is therefore essential to ensure robust methane detection and quantification from time-resolved MTG-FCI observations.

S1.3 Influence on Retrieval Results

To illustrate the impact of co-registration on plume retrievals, Figure S4 shows ΔCH_4 enhancements for the Algeria case on 29 Sep 2023 over 11:08–11:58 UTC. The color bar is fixed to 0–3 ppm, identical to the main text. For each time, the 1st and 3rd columns show unregistered retrievals, while the 2nd and 4th columns show the same scenes after GeFolki registration. Insets zoom into the plume region to focus on structural differences.

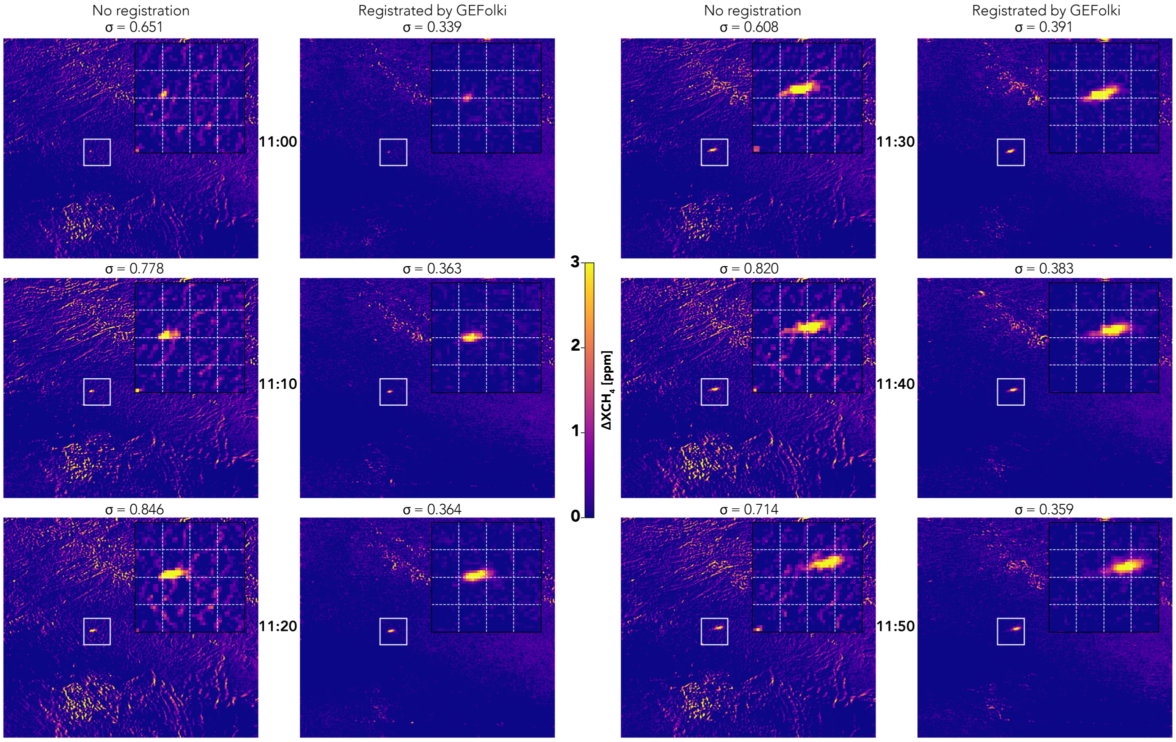

Figure S4: Effect of co-registration on retrieval results for Algeria (29 Sept. 2023 base on 30 Sept. 2023). Colomn 1 & 3: retrievals without correction. Colomn 2 & 4: retrievals after GeFolki registration. Insets show zoomed-in plume regions.

Without registration, the plume appears smeared or distorted, the background exhibits stronger variability. The panel annotations report the background standard deviation (σ) for each scene. Across all times, *unregistered* σ values are around 0.61–0.85 ppm, whereas *registered* σ values reduce to 0.34–0.39 ppm, i.e., a ≈40–60% decrease. Following the convention used for methane detection (Jacob et al., 2016; MacLean et al., 2024), where a methane enhancement should exceed 2σ of the background noise to be deemed detectable, the effective detection threshold is reduced from ∼1.2–1.7 ppm to ∼0.68–0.78 ppm after registration.

These results confirm that sub-pixel misalignments inflate background noise and distort plume morphology even when overall geolocation accuracy is within mission specs. Applying GeFolki stabilizes the background and sharpens the plume, thereby improving the robustness of the retrieved ΔCH_4 fields.

S2 Cloud Screening

Following co-registration, cloud screening was applied to remove pixels contaminated by clouds, which can obscure surface reflectance and interfere with radiative transfer calculations. The cloud ratio (c) is derived from the ratio of IR10.5 (10.5μm) and NIR0.4 (0.4μm), defined as:

$$c = \frac{\text{IR105} - \text{NIR04}}{\text{IR105} + \text{NIR04}} \tag{4}$$

Pixels below the 8th percentile threshold were classified as cloud-covered. The 8th percentile threshold was selected empirically and can be adjusted based on cloud detection performance to optimize the balance between excluding cloudy pixels and preserving clear-sky data. IR105, sensitive to thermal radiation, highlights cold cloud tops, while NIR04, reflective in the near-infrared, enhances cloud-surface contrast. To further ensure cloud contamination was minimized, cloud masks derived from MSG (Meteosat Second Generation) observations were used to validate the selection of cloud-free pixels, as this work was conducted during the commissioning phase of FCI. Superior cloud masking will be available from FCI operational products in future applications.

S3 Uncertainty estimation of IME

The uncertainty of Integrated Methane Enhancement (IME) was assessed by propagating three independent error sources: random noise, plume mask selection, and column height variability. The total uncertainty is expressed as the root-sum-square of the three terms:

$$\sigma_{\mathrm{IME}} = \sqrt{\sigma_{\mathrm{noise}}^2 + \sigma_{\mathrm{mask}}^2 + \sigma_H^2}. \tag{5}$$

Random noise ($\sigma_{\mathbf{noise}}$). The variability of $\Delta X_{\mathrm{CH_4}}$ was estimated from plume-free background pixels as the standard deviation $\sigma_{\Delta X}$. Assuming independent pixel noise, the uncertainty of the integrated methane enhancement (IME) due to random noise can be expressed as:

$$\sigma_{\mathrm{noise}} = M_{\mathrm{CH_4}} \cdot A_{\mathrm{pix}} \cdot \Delta\Omega_{\mathrm{noise}} \cdot \sqrt{N}, \tag{6}$$

where $M_{\mathrm{CH_4}}$ is the molar mass of methane, A_{pix} is the pixel area, and $\Delta\Omega_{\mathrm{noise}}$ is the column enhancement corresponding to $\sigma_{\Delta X}$:

$$\Delta\Omega_{\mathrm{noise}} = \frac{H_{\mathrm{col}} \cdot \sigma_{\Delta X}}{10^6 \cdot V_m}. \tag{7}$$

Here H_{col} is the assumed atmospheric column height and V_m the molar volume of an ideal gas. This formulation propagates the per-pixel concentration variability to IME units and scales with $\sqrt{N}$ to reflect the effect of integrating over all plume pixels.

Mask selection ($\sigma_{\mathbf{mask}}$). To assess the sensitivity of IME to the definition of plume extent, the plume mask was dilated and eroded by one pixel. The corresponding IME values, $\mathrm{IME}_{\mathrm{dilate}}$ and $\mathrm{IME}_{\mathrm{erode}}$, provide upper and lower bounds of the estimate. We define the mask uncertainty as the symmetric half difference:

$$\sigma_{\mathrm{mask}} = \tfrac{1}{2}\left|\mathrm{IME}_{\mathrm{dilate}} - \mathrm{IME}_{\mathrm{erode}}\right|. \tag{8}$$

This approach assumes the original mask represents a best estimate between the two extremes, with the half-range corresponding to a $\pm\sigma$ interval.

Column mass factor (σ_H). We model the sensitivity of the dry-air column to terrain elevation using a barometric scale height $H_s = 8\,\text{km}$. For small perturbations z about the scene mean, the relative change in the dry-air column mass is $\Delta N/N \approx z/H_s$. To approximate the terrain-induced variability, we represent the plume region (20–650 m elevation) by a standard deviation of $\sigma_z \approx 350\,\text{m}$. With a mean column height of $H_{\text{col}} = 8000\,\text{m}$, this yields a fixed relative error of

$$r_H = \sigma_z/H_{\text{col}} \approx 0.044 \quad (4.4\%), \qquad \sigma_H = r_H \cdot \text{IME}. \tag{9}$$

This approach provides a conservative envelope estimate of the DEM-driven contribution to IME uncertainty without requiring pixel-level DEM fields for each retrieval.

These three terms together provide a conservative estimate of IME uncertainty without additional assumptions on pixel geometry or spatial gradients.

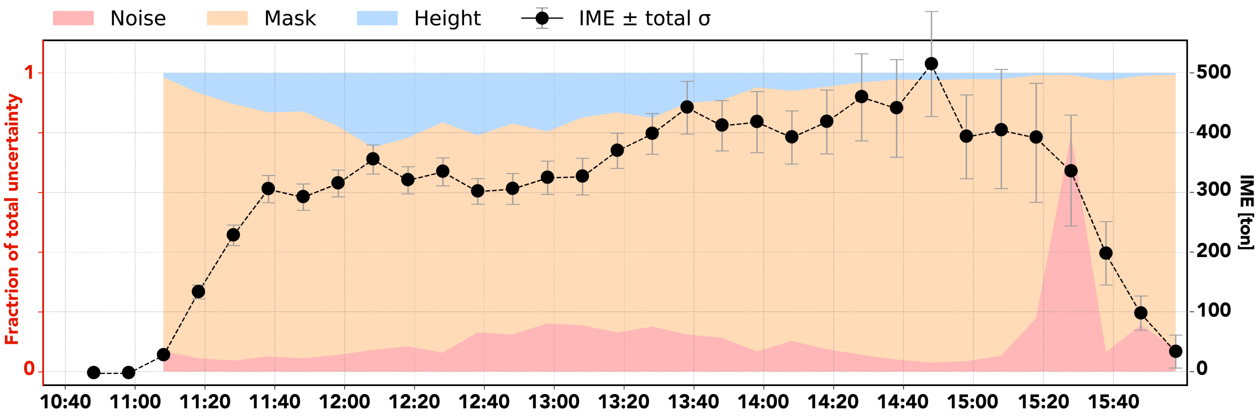

Figure S5: Time series of Integrated Methane Enhancement (IME) with absolute uncertainties (black points, right axis) and fractional contributions of error sources (stacked areas, left axis): random noise (blue), mask selection (orange), and column mass factor (green).

Figure S6 summarizes both the absolute IME values and the fractional contribution of the three error components. Throughout most of the plume evolution, mask selection (σ_{mask}) represents the largest source of uncertainty, reflecting the sensitivity of IME to plume boundary definition. The contribution from random noise (σ_{noise}) remains below ~10% except for a few frames with limited plume extent, while the column mass factor term (σ_H) contributes a nearly constant ~4–5% envelope across the full time series. This analysis points to the critical role of robust mask definition in reducing overall IME uncertainty.

S4 Additional Case

S4.1 Algeria, Oct. 1, 2023

To further investigate the lower detection bound of MTG-FCI, we analyzed the 1 October 2023 release from the same Algerian compressor station, previously reported at ~40–50 t by de Jong et al. (2025) (Figure S6). Retrievals were conducted using 30 September 2023 as the

plume-free reference day at the same local time, ensuring consistent illumination conditions. During the early stage (11:28–11:48 UTC), the plume is clearly identifiable, with per-frame IME values reaching up to ∼65 t. After ∼12:18 UTC, however, the signal weakens to near-threshold levels, making automated threshold-based masking unreliable; plume identification then relies primarily on visual inspection and spatiotemporal coherence in the animation.

The IME time series (panel b) yields a total emitted mass of ∼60 t, corresponding to a mean rate of $\sim 120 \pm 43$ t h^{-1} for an assumed release duration of 30 ± 10 min. This case illustrates that MTG-FCI can marginally detect plumes with per-frame IME of only ∼30-50 t, provided that temporal continuity is exploited, and highlights that detectability is constrained by instantaneous per-frame IME rather than the inferred emission rate.

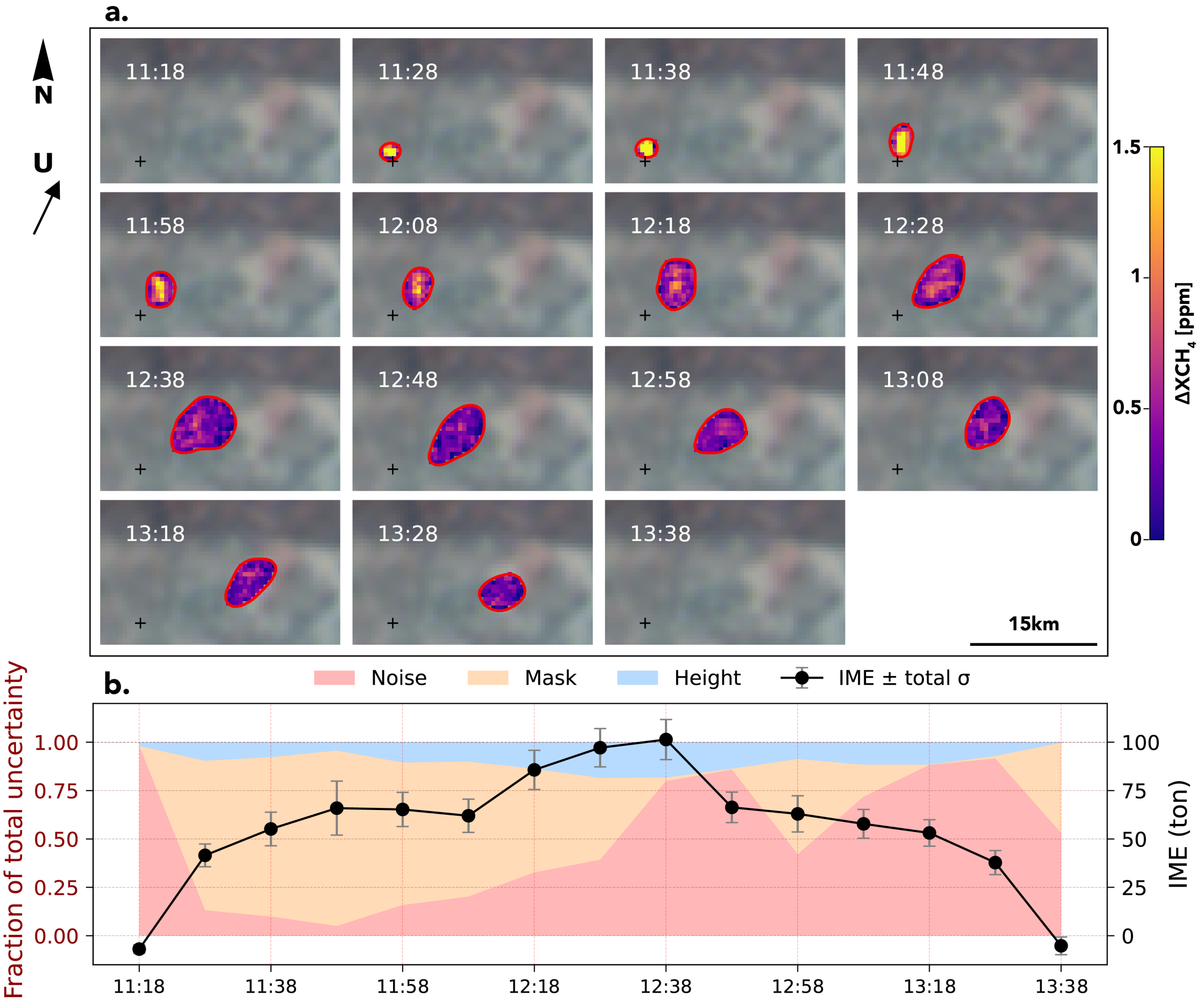

Figure S6: MTG-FCI retrieval of the 1 October 2023 release at the Algerian compressor station (∼50–60 t total mass). Per-frame IME reached up to ∼65 t in the early stage, but later frames weakened to 30–50 t and required spatiotemporal coherence for identification. The total emission is estimated at ∼60 t ($\sim 120 \pm 43$ t h^{-1}).

S4.2 Russia, Mar. 19, 2025 (38.559°E, 50.446°N)

We also examined a plume event from the TROPOMI database located in Russia (Fig. S7, source: `https://www.sron.nl/en/pillars/science/earth/methane/methane-plume-maps/`). Unlike the Algerian examples, this scene is characterized by low surface reflectance and complex topography, resulting in a very low signal-to-noise ratio. According to the SRON weekly report, this event corresponds to an estimated emission rate of 191 ± 63 t h^{-1}, detected at around 10:30 UTC. However, MTG-FCI data are not available during 10:00 and 12:00 UTC this day, leaving a temporal gap that coincides with the SRON detection. This limits the direct comparability of the two observations.

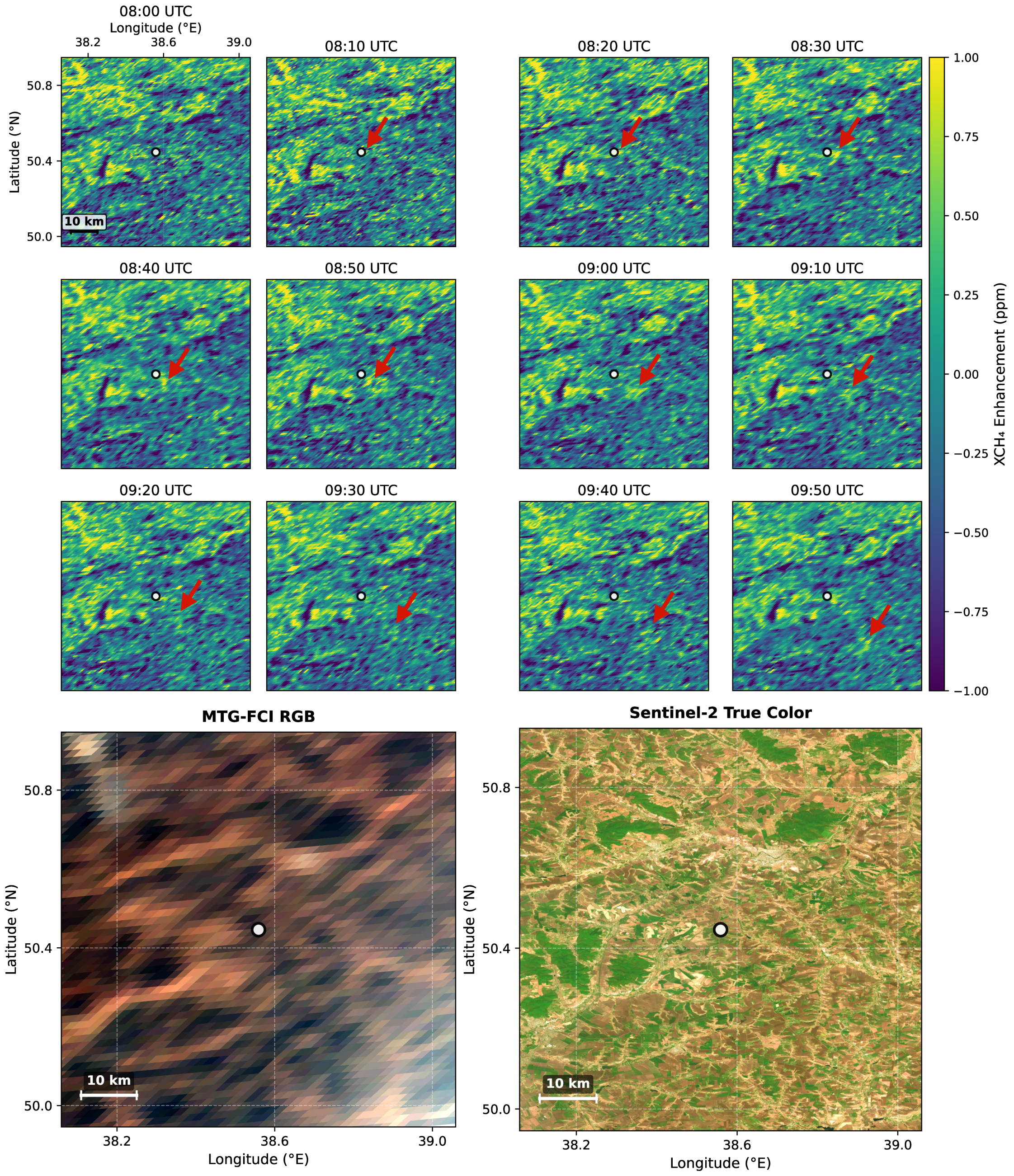

Figure S7: MTG-FCI retrievals of the Russian methane release reported on 19 March 2025 (38.559°E, 50.446°N). Shown are ΔCH_4 enhancement maps from 08:09–09:59 UTC, with arrows marking the reported emission location. Despite an independent emission rate estimate of 191 ± 63 t h^{-1} from the SRON IMEO methane plume database, no distinct plume is discernible in individual MTG-FCI frames; only weak, spatially coherent enhancement patterns appear in the temporal sequence. The inset shows a Sentinel-2 RGB mosaic (March 2025) of the same region, illustrating the dark and heterogeneous surface conditions that likely limit detection sensitivity. The apparent mismatch in field of view between MTG-FCI and Sentinel-2 arises because this site is located close to the edge of the MTG-FCI disk, where projection effects lead to noticeable pixel stretching and geometric distortion.

In the available FCI frames before and after this interval, the methane signal is nearly indistinguishable from background variability. Only when inspecting the full temporal sequence (see Supplementary Animation S1, `https://drive.google.com/file/d/1nKmr4wpn9M4cFVqTM8ltk_IeFZPZqKNb/view?usp=sharing`) does a weak, spatially coherent structure emerge in the downwind direction from the reported source location. Moreover, the reference background for this case was selected from March 7, 2025, due to the lack of nearby cloud-free days. The 12-day temporal mismatch likely introduced additional variability in the radiance ratio baseline, further contributing to noisy retrievals. A simple estimation of the Q_{min} framework using local wind speed conditions obtained from ERA5 hourly product yields a lowest detection threshold of $\sim$224 t h^{-1}, which is notably higher than the SRON-reported 191 ± 63 t h^{-1}.

Appendix

Table S4: MTG-FCI acquisition dates used for Q_{min} estimation. All observations were acquired at 13:18 UTC.

**Target Date**	**Reference Date**
2024-10-01	2024-10-02
2024-10-10	2024-10-11
2024-10-20	2024-10-21
2024-11-01	2024-11-02
2024-11-10	2024-11-11
2024-11-22	2024-11-21
2024-12-01	2024-12-02
2024-12-12	2024-12-11
2024-12-20	2024-12-21
2025-01-01	2025-01-02
2025-01-10	2025-01-11
2025-01-20	2025-01-19
2025-02-01	2025-02-02

References

Aksakal, S. K. Geometric accuracy investigations of SEVIRI high resolution visible (HRV) level 1.5 Imagery. *Remote Sensing* **2013**, *5*, 2475–2491.

Aplyer GeFolki: Optical Flow based image registration. `https://github.com/aplyer/gefolki`, Accessed: September 16, 2024.

Plyer, A.; Colin-Koeniguer, E.; Weissgerber, F. A new coregistration algorithm for recent applications on urban SAR images. *IEEE Geoscience and Remote Sensing Letters* **2015**, *12*, 2198–2202.

Brigot, G.; Colin-Koeniguer, E.; Plyer, A.; Janez, F. Adaptation and evaluation of an optical flow method applied to coregistration of forest remote sensing images. *IEEE Journal of Selected Topics in Applied Earth Observations and Remote Sensing* **2016**, *9*, 2923–2939.

Debaecker, V.; Kocaman, S.; Saunier, S.; Garcia, K.; Bas, S.; Just, D. On the geometric accuracy and stability of MSG SEVIRI images. *Atmospheric Environment* **2021**, *262*, 118645.

Yamamoto, Y.; Ichii, K.; Higuchi, A.; Takenaka, H. Geolocation accuracy assessment of Himawari-8/AHI imagery for application to terrestrial monitoring. *Remote Sensing* **2020**, *12*, 1372.

Jacob, D. J.; Turner, A. J.; Maasakkers, J. D.; Sheng, J.; Sun, K.; Liu, X.; Chance, K.; Aben, I.; McKeever, J.; Frankenberg, C. Satellite observations of atmospheric methane and their value for quantifying methane emissions. *Atmospheric Chemistry and Physics* **2016**, *16*, 14371–14396.

MacLean, J.-P. W.; Girard, M.; Jervis, D.; Marshall, D.; McKeever, J.; Ramier, A.; Strupler, M.; Tarrant, E.; Young, D. Offshore methane detection and quantification from space using sun glint measurements with the GHGSat constellation. *Atmospheric Measurement Techniques* **2024**, *17*, 863–874.

de Jong, T. A.; Maasakkers, J. D.; Irakulis-Loitxate, I.; Randles, C. A.; Tol, P.; Aben, I. Daily global methane super-emitter detection and source identification with sub-daily tracking. *Geophysical Research Letters* **2025**, *52*, e2024GL111824.
